# Immunomodulatory biomaterials for implant-associated infections: from conventional to advanced therapeutic strategies

**DOI:** 10.1186/s40824-022-00326-x

**Published:** 2022-12-05

**Authors:** Jiale Dong, Wenzhi Wang, Wei Zhou, Siming Zhang, Meng Li, Ning Li, Guoqing Pan, Xianzuo Zhang, Jiaxiang Bai, Chen Zhu

**Affiliations:** 1 Department of Orthopedic Surgery, The First Affiliated Hospital of University of Science and Technology of China, Anhui Provincial Hospital, 230001 Hefei, Anhui P. R. China; 2 Medical College, Soochow University, 215006 Suzhou, Jiangsu P. R. China; 3 Institute for Advanced Materials, School of Materials Science and Engineering, Jiangsu University, 212013 Zhenjiang, China

**Keywords:** Implant-associated infections, Immune modulation, Biomaterials, Antibacterial therapeutic strategies

## Abstract

Implant-associated infection (IAI) is increasingly emerging as a serious threat with the massive application of biomaterials. Bacteria attached to the surface of implants are often difficult to remove and exhibit high resistance to bactericides. In the quest for novel antimicrobial strategies, conventional antimicrobial materials often fail to exert their function because they tend to focus on direct bactericidal activity while neglecting the modulation of immune systems. The inflammatory response induced by host immune cells was thought to be a detrimental force impeding wound healing. However, the immune system has recently received increasing attention as a vital player in the host’s defense against infection. Anti-infective strategies based on the modulation of host immune defenses are emerging as a field of interest. This review explains the importance of the immune system in combating infections and describes current advanced immune-enhanced anti-infection strategies. First, the characteristics of traditional/conventional implant biomaterials and the reasons for the difficulty of bacterial clearance in IAI were reviewed. Second, the importance of immune cells in the battle against bacteria is elucidated. Then, we discuss how to design biomaterials that activate the defense function of immune cells to enhance the antimicrobial potential. Based on the key premise of restoring proper host-protective immunity, varying advanced immune-enhanced antimicrobial strategies were discussed. Finally, current issues and perspectives in this field were offered. This review will provide scientific guidance to enhance the development of advanced anti-infective biomaterials.

## Introduction

With the aging population and the rising demand for quality of life, a growing number of biomedical implants are applied every year [1]. However, implant-associated infection (IAI) is increasingly emerging as a serious threat with the widespread use of biomaterials. Indeed, more than a quarter of healthcare-associated infections are related to medical devices in the US [2]. IAI directly leads to the failure of the surgery, and patients will subsequently suffer from great pain and huge expenses in the following treatment [3]. Therefore, the prevention of IAI is of top priority in implant replacement surgeries.

Generally, traditional implant biomaterials do not possess ideal antibacterial activity [4], so antibiotics are routinely applied to prevent possible postoperative infections after implantation [5]. However, regional subinhibitory antibiotic concentrations and intermittent antibiotic exposure may exacerbate the selection of drug-resistant mutant strains, the most representative of which is the emergence of methicillin-resistant *Staphylococcus aureus* (MRSA) [6]. Besides, antibiotics are ineffective for established biofilms on implants, because biofilms can facilitate bacterial resistance to the harsh physicochemical environment and block antibiotic penetration and killing [7].

The immune system is a powerful shield of the body against pathogens. It responds rapidly (within hours), recognizes pathogens nonspecifically, and attempts to kill invaders through various mechanisms [8]. However, implant biomaterials often interfere with the normal function of immune cells [9, 10]. For example, neutrophils produced excess reactive oxygen species (ROS) and died rapidly when exposed to polytetrafluoroethylene and dacron in vascular biomaterials [11], indicating an unfavorable immunosuppressive effect on the microorganism killing around the implant. For macrophages, implants can reduce their phagocytic ability and promote their fusion into foreign body giant cells (FBGCs), which are the foundation of forming a dense fibrous layer, hindering long-term biomaterial-host integration. In addition, the induction of macrophages by bacteria biofilm also disrupts the normal M1/M2 phenotypic transition, reduces the killing effect of macrophages, and leads to chronic infections [12, 13].

Previously, various anti-infective strategies one-sidedly focused on killing pathogens, including designing antifouling coatings on biomaterial surfaces to reduce bacterial adhesion or loading antimicrobial drugs to actively kill bacteria [14, 15]. However, these strategies showed doubtful efficacy in treating IAI. It is unrealistic to avoid all bacterial adhesion with simple antifouling coatings, and the coatings will likewise impede host cell adhesion and thus slow the healing process. Although some biomaterial designs claim to be able to clear the vast majority of the bacteria, the residual bacteria may still cause the recurrence of infection and contribute to chronicity. Unfortunately, it is found that some bacteria, such as *S. aureus*, can internalize into nonspecific immune cells to evade the killing effect of antibacterial drugs [16, 17], and current direct antimicrobial strategies are powerless against the bacteria in these “Trojan horse cells.” Given this, it becomes equally important to restore or potentiate the antibacterial activity of the normal immune system. The clearance of residual bacteria and the targeted killing of bacteria-infected cells rely on various immune cells. Interestingly, some materials, such as antimicrobial peptides and metal ions, have not only been found to possess intrinsic antibacterial activity but also the ability to modulate immune cells, suggesting a potential direction for designing novel strategies against IAI in the future.

In the present review, we describe the characteristics of traditional implanted biomaterials and discuss the mechanisms of antibiotic resistance. We focus on implanted biomaterials in orthopedics because these implants have a long and even permanent retention time in the body. The diagnosis and treatment of IAI in orthopedics are more difficult and can result in severe consequences such as amputation. Compared to general surgical infections, orthopedic surgical infections require a longer interval before the second surgery, which prolongs patient suffering. Nonetheless, other medical implants follow similar infection mechanisms. Then, we highlight the critical role of immune cells in combating infections. We detail the functions of immune cells and the mechanisms by which they are suppressed in IAI. Based on the key premise of restoring proper host-protective immunity, we subsequently discuss immune-enhanced antimicrobial strategies in conventional and advanced treatment strategies. Such novel strategies strive to achieve the combination of inherent antibacterial capacity and efficient immunomodulation, which are critical to promoting pathogen clearance and subsequent healing. We also discuss smart delivery strategies for antimicrobial agents, as deviations in drug delivery targets and dosage control may lead to unpredicted off-target effects and thus counteract the original antimicrobial advantage. In the last part, we evaluate current issues and future directions in the exploration of immune-enhanced antimicrobial strategies.

## Implanted biomaterials

**Fig. 1:**
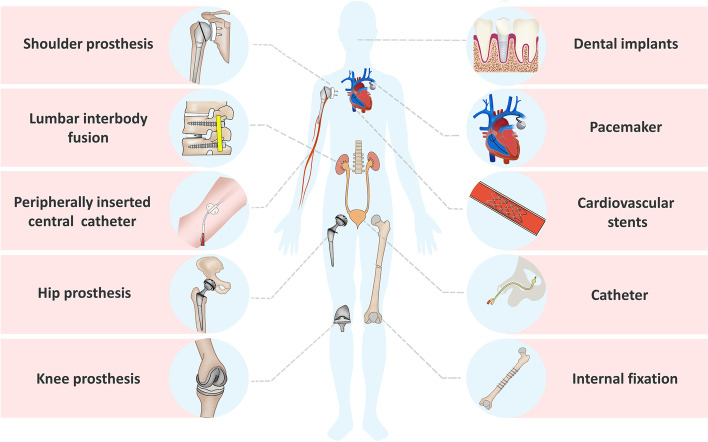
Schematic illustration of common medical devices. Various biomaterials are widely used in the manufacture of medical devices, such as artificial joints, dental implants, cardiovascular stents, pacemakers, various medical catheters and internal fixation apparatuses. They are used directly or indirectly in the human body for the diagnosis and treatment of diseases or to compensate for the function of impaired organs. However, device-associated infections are increasingly emerging as a serious threat with the massive application of biomaterials

Biomaterials have been developed for over a hundred years since Gluck proposed artificial implants in the late 19th century [18]. Implant materials can be divided into metallic and non-metallic materials. They are widely used in the manufacture of medical devices, such as cardiovascular stents, pacemakers, artificial joints, dental implants, and various medical catheters (Fig. 1). Biomaterials used in orthopedics require good mechanical properties, wear resistance, and biocompatibility, and those under development also emphasize controlled biodegradability, excellent bioactivity, and anti-infective capacity.

### Metal biomaterials

Metals are the most widely used implant biomaterials in clinical practice due to their excellent mechanical properties, plasticity, and biological inertness. Stainless steel was the metal material used earliest in the medical field [19]. It is an alloy containing iron, chromium, nickel, and other elements, which exhibits corrosion resistance because chromium can form an oxide film on the surface of the alloy to prevent continued oxidation. However, this advantage no longer exists under physiological conditions, and chloride ions rich in body fluids can easily corrode stainless steel and greatly reduce its fatigue resistance [20]. Cobalt-chromium and titanium alloys have better corrosion resistance and gradually replace stainless steel as long-term implant materials [21, 22]. Nevertheless, the elastic modulus of cobalt-chromium alloys does not match that of human bone. Alloys with a larger elastic modulus will carry more stress, i.e., stress shielding. The stress shielding effect promotes the development of bone resorption around the implant, which is not conducive to the long-term survival of the implant [23]. Despite the relatively low modulus of elasticity of titanium alloys, their poor wear resistance and flexural strength make the reliability of titanium alloys as long-term implants questionable.

Another unavoidable concern with metallic materials is the exudation of metal ions. Cobalt, chromium, and nickel plasmas are considered toxic [24, 25]. Despite the bioinertness of titanium-based implants and their presumed high corrosion resistance, their wide implantation comes with continuous safety concern due to metal dissolution. Ti-6Al-4 V, a commonly used titanium alloy material, dissolves V and Al ions that may lead to the pathogenesis of neuropathy and Alzheimer’s disease [26, 27]. In addition, Ti particles and degradation products of titanium have been detected in the tissues around implants in multiple studies, especially around dental implants, which may be related to biocorrosion, mechanical wear and interaction with substances produced by inflammatory cells or adherent biofilms [28, 29]. In the case of peri-implantitis, bacterial products (such as LPS and acid) and the accompanying inflammation exacerbate erosion, leading to more severe Ti particle release [28, 30]. Foreign body reactions triggered by these particles impair local immune defense due to excessive exhaustion of host immune cells (such as macrophages). Meanwhile, Ti particles lead to sustained activation of the inflammatory response and the release of pro-inflammatory cytokines (such as IL-1β, TNF-α and RANKL), which is obviously detrimental to the osseointegration of biomaterials [31]. Eventually, this vicious circle inevitably leads to implant failure. In light of this, it is necessary to improve the safety and antibacterial ability of metal biomaterials.

### Non-metallic biomaterials

Non-metallic biomaterials include bioceramics, bioactive glass, and various polymer materials. These materials are widely used in the medical field, involving the manufacture of various artificial organs and medical devices, such as artificial teeth, artificial bones, joints, artificial eyes, artificial heart valves, venous cannulae, urinary catheters, etc. These materials also generally lack antibacterial and biological activities. Many biomaterials (such as hernia patches and silicone prostheses) often lead to surgical failure due to uncontrolled device-associated infection. Polytetrafluoroethylene (PTFE), a fluorinated implant biomaterial commonly used in cardiovascular reconstruction, hernia repair, and cosmetic and reconstructive surgery, has a porous microstructure that facilitates bacterial harborage and predisposes to infection [32, 33]. Small particles of some polymer materials (such as polyethylene tear debris and polymethyl methacrylate cement particles) often lead to adverse inflammatory responses around the implant [34, 35]. Much effort is being made to enhance the antibacterial and positive immunomodulatory capabilities of these materials.

## Implant-associated infection

Implant-associated infection, as one of the most frequent and severe complications affecting implant surgery, is characterized by pain, swelling, and loss of function. Subsequent surgery is often required to remove the impaired implants. Biomaterials are implanted in almost any anatomical location in human bodies for medical use, and they interface with various human tissues. The implanted grafts can compensate or replace the function of damaged organs, but they are still foreign bodies that may disturb the immune microenvironment. Even a slight tissue response can disturb the immune defense at the implant site, creating a locus minoris resistentiae susceptible to bacterial attack even by opportunistic bacteria with weak virulence. In orthopedics, IAI can be classified as early and late infections. Infections that occur within 1 month postoperatively are classified as early infections, while late infections develop after more than 1 month, which is usually insidious and difficult to diagnose accurately [36]. Although most IAI requires surgical treatments, the decision to preserve the implants depends heavily on the chronicity of the infection because debridement and prosthesis retention are only effective for early infections. Gram-positive cocci are the most commonly isolated microorganisms, particularly *Staphylococcus aureus* (*S. aureus*) and *Staphylococcus epidermidis* (*S. epidermidis*) [37]. *S. aureus* is often associated with acute implant infections, while *S. epidermidis* often causes late chronic infections. Mixed infections (simultaneous infection with two or more microorganisms) often occur after sinus tract formation, which exacerbates the difficulty in treatment.

Multiple factors are involved in postoperative infections, including individual patient factors, local prosthetic factors, bacterial virulence, and number. To prevent infection, it is necessary to correct the patient’s underlying diseases (such as anemia and diabetes) preoperatively and to ensure a sterile intraoperative surgical environment. Prophylactic application of antibiotics is also routinely used to reduce postoperative infections. Common antibiotics used in the perioperative period include cephalosporins, rifampin, gentamicin, and vancomycin. These antibiotics kill bacteria by interfering with the synthesis of the cell wall or other bacterial constituents. For example, rifampin interferes with nucleic acid synthesis, and gentamicin inhibits bacterial protein synthesis by binding to the 30 S subunit of the ribosome. Cephalosporins and vancomycin kill bacteria by disrupting their cell walls [38]. Such single-target antimicrobial mechanism of these drugs implies a greater risk of antibiotic resistance. Therefore, multiple antibiotics are often used in combination [39–41]. However, the incidence of IAI has not decreased with these preparations for surgery. Bacterial virulence and drug resistance are still increasing. Up to 40% of *S. epidermidis* and 32% of *S. aureus* strains isolated from orthopedic IAI have been reported to be resistant to gentamicin [42–44].

### The threat of bacterial antimicrobial resistance

Growing bacterial antibiotic resistance makes the prevention and treatment of IAI increasingly difficult. This resistance may be intrinsic or acquired [45]. The intrinsic antibiotic resistance of bacteria may arise from differences in the bacterial structure. For example, vancomycin has superior antibacterial activity against Gram-positive bacteria by interfering with cell wall synthesis, whereas the permeation barrier of the outer membrane of Gram-negative bacteria prevents its action [46].

In addition to intrinsic antibiotic resistance, bacteria can develop or acquire resistance through genetic mutations and horizontal gene transfer (HGT). This acquired resistance does not depend on structures specific to some bacterial species and is more harmful. Especially for IAI, because the biofilms attached to the implants contain a huge bacterial load, in which bacteria are more likely to undergo genetic mutations and HGT. The mechanisms of acquired bacterial resistance can be classified into three categories [45]. The first mechanism is to minimize the intracytoplasmic concentration of antibiotics by reducing infiltration or increasing efflux. These procedures involve either downregulation of the number and activity of porin proteins for antibiotic diffusion on the bacterial surface or overexpression of the bacterial efflux pump. Some bacterial antibiotic efflux pumps are specific for one antibiotic, but nonspecific efflux pumps often mediate bacterial resistance to multiple antibiotics. The second mechanism of resistance is to alter the target proteins recognized by antibiotics. Mutation or recombination of bacterial target genes to provide mosaic alleles leads to the reduced affinity of the functional target proteins for the antibiotic. Another method to protect target proteins is target modification. Although not altering the primary protein sequence, bacteria can modify the target by adding chemical groups to prevent antibiotic binding. The third mechanism is to modify or protect antibiotics directly. Bacteria produce a range of enzymes to chemically modify the structure of antibiotics through acylation, phosphorylation, or glycosylation, making the antibiotics unable to bind to their target proteins. Bacteria can also directly produce hydrolases to destroy antibiotics. The first β-lactamase produced by bacteria was discovered in 1940 [47]. Subsequently, a number of enzymes were found to degrade different types of antibiotics, including β-lactams, macrolides, and aminoglycosides.

Bacterial gene mutations and HGT promote the rapid emergence and spread of drug resistance genes. On the one hand, the application of antibiotics kills most sensitive bacteria, and the proliferation of a few resistant mutants leads to increased resistance to antibiotics. On the other hand, some drug resistance genes encoded on bacterial chromosomes can be transferred to plasmids or phages. These mobile genetic progenitors carrying resistance genes are transferable among different bacteria. This makes it possible for resistance mechanisms generated by one pathogen to rapidly spread to other clinically relevant pathogens. For example, since the report of the resistance gene of new Delhi metallo-β-lactamase-1 (a metallo-β-lactamase that hydrolyzes all β-lactams except aztreonam) in a Klebsiella strain in 2008, the gene has been found within a variety of bacteria and has spread widely around the world [48, 49].

### Formation of biofilms

**Fig. 2:**
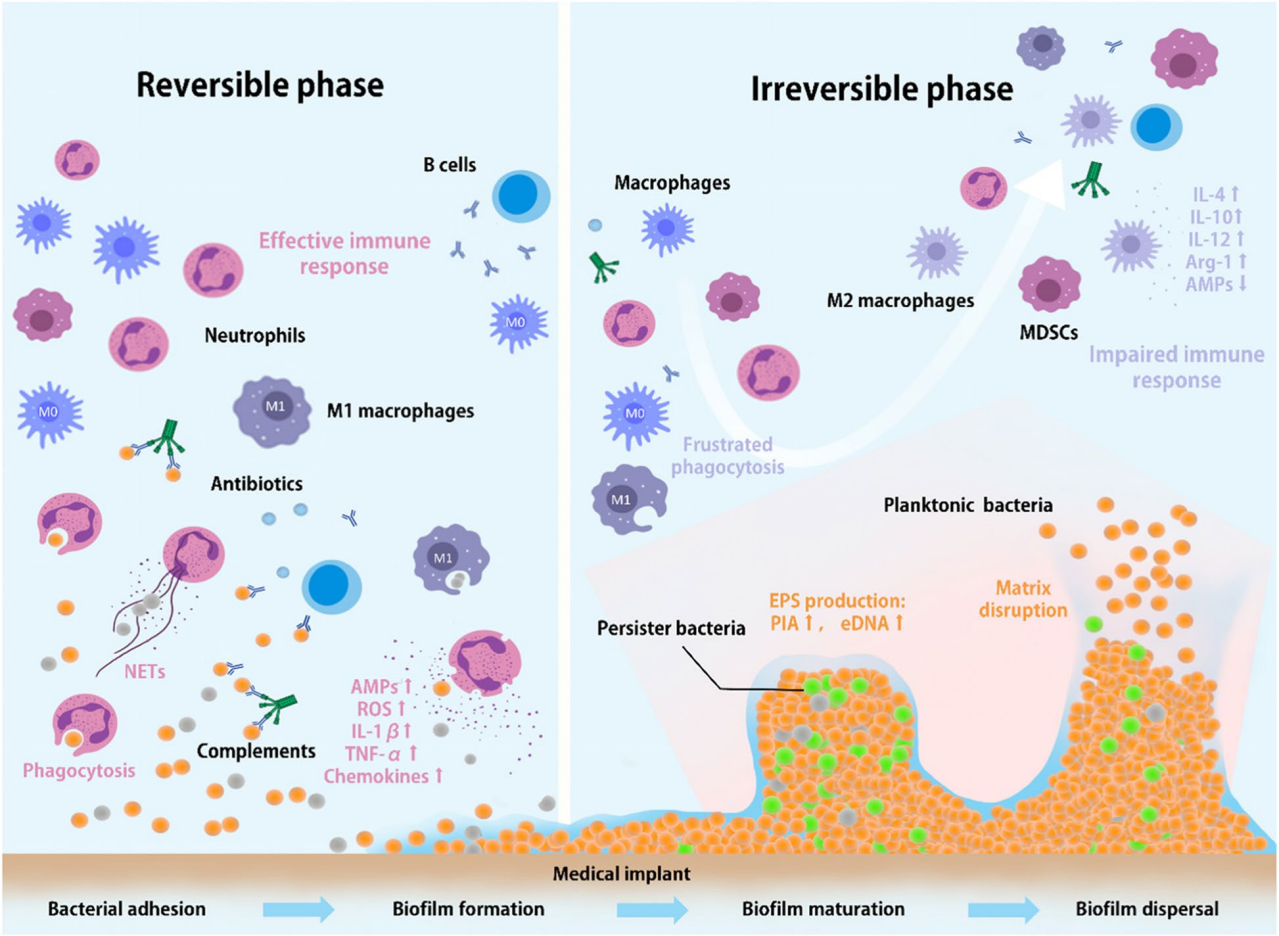
Response of host immune cells during different phases of biofilm formation. IAI can be divided into reversible and irreversible phases. This is closely related to the time interval of bacterial colonization on the implants. In the early stages of bacterial adhesion, innate immune cells can effectively kill bacteria by phagocytosis, oxidative bursts, production of antimicrobial peptides (AMPs), formation of neutrophil extracellular traps (NETs) and secretion of pro-inflammatory cytokines (such as IL-1β, IL-6 and TNF-α) and chemokines (such as MCP-1 and CXCL1). Because of the susceptibility of bacteria to clearance by host immune cells at this stage, it is considered a window for effective prevention of IAI. Biofilms mature gradually as bacteria accumulate and produce extracellular polymeric substances (EPSs, such as PIA and eDNA), which not only hinder the penetration and attack of antibiotics and immune cells but also skew the local immune response toward the anti-inflammatory type and suppress the host defense system. For instance, biofilms induce macrophage polarization from the classic M1 toward the M2 phenotype, which is characterized by the increased expression of anti-inflammatory cytokines (such as IL-4, IL-10, IL-12 and Arg-1) and attenuated antimicrobial peptide production. Simultaneously, they modulate the excessive expression of myeloid-derived suppressor cells (MDSCs, a population of immature myeloid cells mainly exerting strong immunosuppressive effects). The formation of mature biofilms marks the irreversible stage of infection. When bacteria within a mature biofilm reach a certain number, they will be dispersed and migrate to a new site to form new biofilms. Dispersal is facilitated by enzymatic degradation of surfactant molecules and EPSs and inhibition of biofilm matrix production. The quorum sensing system regulates the production of degrading enzymes in a density-dependent manner

Biofilm formation is one of the most challenging problems in combating infections because they usually form in a very short period and are difficult to remove completely. For example, *Staphylococcus aureus*, which is common in implant-associated infections, can form biofilms within three hours (Fig. 2) [50]. Biofilms are bacterial-aggregated communities composed of bacteria and their secreted extracellular polymeric substances (EPSs), including polysaccharides, proteins, extracellular DNA (eDNA), etc. They not only facilitate bacterial resistance to the harsh environment but also block the penetration of antibiotics and the killing effect of immune cells [13]. In addition, communication between bacteria within biofilms often allows them to acquire greater resistance and virulence. Biofilms contribute to the persistence of IAI. In this section, we focus on the process of bacterial biofilm formation.

#### Bacterial adhesion

Bacterial adhesion is the first step in the development of IAI. Bacterial adhesion can be divided into two stages. The first stage is the accidental encounter of bacteria with the implant surface when bacteria deposit on the prosthesis surface under nonspecific forces such as van der Waals forces and electrostatic interactions. Due to the loose binding of bacteria to the surface, bacterial adhesion in this phase is reversible. Antibiotic intervention and debridement surgery in a timely manner can achieve bacterial eradication. However, as the bacterial adhesins gradually bind to the matrix proteins on the prosthesis surface in the second stage, bacteria are firmly anchored to the prosthesis and the adhesion process becomes irreversible [17, 51, 52].

After implantation, extracellular matrix proteins such as fibronectin and hyaluronan rapidly cover the prosthesis surface. Deposition of these proteins can provide anchors for host cell adhesion. By binding to integrin, the host cells adhere to the surface of the biomaterial. For instance, integrins β1 and β2 mediate adhesion and facilitate the motility and phagocytosis of macrophages. Osteoblast precursor cells also spread on the biomaterial surface via integrin, which is essential for the osseointegration of the prosthesis. However, bacteria can also achieve a similar adhesion process by expressing adhesins. *S. aureus* expresses microbial surface components recognizing adhesive matrix molecules (MSCRAMMs) to promote adhesion [53]. The pili and pilus-like structures on the surface of Gram-negative and some Gram-positive bacteria (such as *Actinomyces naeslundii* and *Streptococcus parasanguis*) also play a role as adhesins [54, 55]. Therefore, the adhesion of bacteria to the prosthesis surface competes with that of host cells, with the first adhered and colonized cells gaining a greater advantage and preventing the adhesion of competitors [6, 56]. In addition, the disruption of the local blood supply due to surgical trauma leads to an immunosuppressive microenvironment that promotes bacterial colonization and reduces the required bacterial number for infection. In a rat osteomyelitis model, a simple dose of 100 CFUs of *S. aureus* was sufficient to induce orthopedic IAI [57].

#### Biofilm formation

Upon adhesion, bacteria secrete various substances, including proteins, extracellular polysaccharides, and eDNA, to form biofilms [7]. Polysaccharides are the main components of the biofilm matrix and are essential for bacterial resistance to environmental stress and immune cells. The polysaccharide intercellular adhesin (PIA), encoded by the icaADBC locus, is the major polysaccharide of the biofilm matrix [58]. Harsh environments, such as exposure to heat, ethanol, and high osmolarity, greatly promote PIA production. Bacteria that produce PIA have been reported to possess higher antibiotic resistance. eDNA is mainly produced by altruistic bacterial suicide or fratricide killing [59]. It is essential for maintaining the stability of biofilms, facilitating bacterial HGT, and regulating host immune responses. The proteins in biofilms not only promote strong adhesion of bacteria to biomaterial surfaces but also have other roles, such as facilitating intercellular communication, acting as bacterial virulence factors, and interfering with host immune responses.

Biofilms contribute to bacterial resistance to antibiotics. Compared to planktonic bacteria, biofilms can increase the minimum inhibitory concentration of antibiotics up to 1000-fold [60–62]. Several mechanisms may explain the potent resistance effect of biofilms. First, the thick biofilm matrix prevents the penetration of antibiotics. Second, the hydrolytic enzymes produced by bacteria can accumulate to higher concentrations within the biofilms to destroy antibiotics. Third, the huge bacterial load within biofilms poses more difficulties for eradicating these bacteria. Frequent HGT between bacteria also enables the rapid development of drug resistance. Besides, the development of antibiotic persister mutants in biofilms is one of the reasons for the poor efficacy of antibiotics, which may be related to the stringent response triggered by bacteria under environmental stress [63].

Biofilms can likewise help bacterial defense against invasions from immune cells. Neutrophils and macrophages are the main effector cells of the innate immune system, mediating phagocytosis and killing pathogens. Biofilms resist penetration and phagocytosis of these cells. Although neutrophils can phagocytose scattered bacteria effectively, they are powerless against bacteria that live within biofilms. Kovach et al. found that the elastic modulus of biofilms is greater than the pressure exerted by neutrophils during phagocytosis (less than 1 kPa) [64], which prevents neutrophils from tearing the biofilm into smaller fragments. Thus, biofilms provide mechanical protection for bacteria against phagocytic clearance [6]. Macrophages also face similar dilemmas [7]. Moreover, the immune microenvironment surrounding the biofilm induces macrophages to polarize from a pro-inflammatory (M1) to an anti-inflammatory phenotype (M2), thereby assisting the evasion of clearance of infection.

#### Biofilm dispersal

When bacteria within a mature biofilm reach a certain number, they will be released and migrate to a new site to form a new biofilm. This process is facilitated by the inhibition of polysaccharide and relative proteins production and degradation of the biofilm matrix by various enzymes, such as phenolsoluble modulins proteases and nucleases. Biofilm dispersal often leads to an increased inflammatory response causing systemic symptoms. It can also delay the course of infection and increase the difficulty of anti-infective treatment.

The quorum sensing (QS) system, a communication pathway between microbial cells, plays a critical role in the dispersal process [65, 66]. Within biofilms, bacteria communicate with each other by synthesizing autoinducer (AI). The extracellular concentration of AI increases correspondingly with increasing bacterial density. Therefore, bacteria can monitor changes in their population densities based on AI in the surrounding environment. Once the concentration of AI reaches a certain threshold, the expression of related genes in bacteria will be initiated and regulate the biological behavior of the bacteria. Different bacteria have different AIs, with Gram-negative bacteria generally using N-acylated homoserine lactone-type molecules as AI and Gram-positive bacteria using autoinducer peptides. Some bacteria even use two or three different signaling molecules to regulate their population behavior, which also shows the complexity of the QS mechanism. AI-2 is a universal signaling molecule produced by many Gram-negative and Gram-positive bacteria. In the dispersal phase, the quorum sensing system regulates the production of degrading enzymes in a density-dependent manner. In addition, the QS system is of great significance in inducing biofilm formation and activating bacterial virulence.

### Immune evasion of bacteria

Some bacteria species (such as *P. aeruginosa* and *S. aureus*) can hide in host cells to evade host defenses and antibiotics [17]. For example, fibronectin-binding protein (FnBP, one of the MSCRAMMs) expressed by *S. aureus* can bind to fibronectin of osteoblasts and promote bacterial adhesion and subsequent internalization. This infection process induces a high expression of tumor necrosis factor-related apoptosis-inducing ligand (TRAIL) and eventually leads to osteoclast apoptosis through activation of Caspase-8 [67]. Part of *S. aureus* grows slowly and secretes low levels of cytotoxic factors, known as small colony variants [68]. These variants can survive in host cells for a long time, causing chronic implant infections.

## The role of immune cells in antibacterial activity with biomaterials

The innate immune system, which involves the joint participation of a series of immune cells, plays a pivotal role in the conventional antibacterial mechanism (Fig. 2). Currently, even the most biocompatible implants are considered foreign to the body, which are considered harmful and are the targets for immune attacks. This misidentification often leads to a significant depletion of immune cells followed by a reduced bactericidal capacity. The chronic inflammation caused by foreign body reactions often prevents wound healing. These processes involve a series of interactions with immune cells and biomaterials. The ideal biomaterial should be capable of modulating the immune response to help eradicate bacteria or accelerate tissue healing, which requires an in-depth understanding of the function of immune cells. In this chapter, we explore the role of individual immune cells in antimicrobial activity with biomaterials in detail.

### Macrophages

Macrophages are dynamic cells whose polarization can be affected by different stimuli. Polarized macrophages can be broadly divided into two phenotypes: pro-inflammatory M1-type macrophages and anti-inflammatory M2-type macrophages. M1-type macrophages promote inflammatory responses against bacteria, while M2-type macrophages inhibit inflammation and cause chronic bacterial infections. Nonetheless, M2-type macrophages are essential in tissue repair in the late stages of inflammation. The transition between M1 and M2 phenotypes of macrophages in vivo is a continuous and complex process as macrophages are involved in the dynamic regulation of the promotion and resolution of inflammatory response after pathogen infection [69, 70].

In the presence of implants, cytokines released by neutrophils and mast cells mediate the recruitment of macrophages to the vicinity of the implant [71]. M1-type macrophages participate in the inflammatory response to remove necrotic tissue and cell debris generated during biomaterial implantation [51]. In the tissue defect site, M2-type macrophages participate in tissue repair. The failed macrophage transition from M1 to M2 can impede the wound healing process. Furthermore, biomaterials with poor biocompatibility in this condition will lead to the fusion of membranes and the formation of FBGCs, indicating the formation of chronic inflammation. FBGCs are long-lasting and closely associated with the formation of fibrous membranes that isolate the implants from the tissue, which may cause implant failure eventually. In addition, FBGCs exhibit reduced bactericidal ability, in particular, reduced production of bactericidal substances and decreased phagocytic capacity [10]. Therefore, regulating the normal polarization transition of macrophages and preventing the formation of FBGCs are crucial for the long-term survival of implants [71, 72].

When a pathogen enters the host, the antigen stimulates macrophages to activate and remove the pathogen from the host. M1-type macrophages play a pivotal role in resistance to bacteria during acute infection, which is associated with the secretion of antimicrobial substances, stronger phagocytic function, and antigen presentation of M1 macrophages. However, some pathogens have also evolved different strategies to interfere with the polarization of macrophages and become difficult to remove. For example, some bacteria interfere with the immune response by downregulating NF-κB and stimulating IL-10 expression [73]. *S. aureus*, as the most common bacterium in orthopedic IAI, can mediate macrophage polarization toward the M2 phenotype. Long-term chronic infection of pathogens is considered to be related to the M2-type polarization of macrophages. When staphylococcal biofilms are formed on the implant surface, the bactericidal activity of macrophages is inhibited, the phagocytosis and killing of bacteria are weakened, and bacteria cannot be removed effectively [51].

### Neutrophils

Neutrophils are immune cells that reach the infected site immediately after pathogen invasion. They clear pathogens in acute infection through a variety of mechanisms. The first mechanism is to phagocytize pathogens and utilize ROS or antimicrobial proteins to remove pathogens. The second is the release of antimicrobial proteins from neutrophils to eliminate pathogens. Cationic antimicrobial peptides exert antibacterial effects by binding to anions on the bacterial surface to destroy cell membranes [74]. The third is the removal of pathogens by highly activated neutrophils through the release of neutrophil extracellular traps (NETs) [75]. Neutrophils make important ‘anti-pathogen decisions’ based on the size and type of microorganisms when reaching the inflammatory site. The corresponding anti-pathogen strategy is determined to effectively remove pathogens while minimizing damage to the host [76].

Neutrophils mature in the bone marrow and are released into the blood. They are the most abundant innate immune cells in the blood. In the presence of an implant, the concentration of neutrophils rises sharply and is released around the implant. Neutrophils persist around implants and NETs may deposit around some implants [77–79]. The long-term presence of NETs can cause damage to normal tissue. Therefore, it is extremely important to regulate the number of neutrophils on the implant surface when designing implants [51].

*S. aureus* is the leading cause of infection, and MRSA, which is associated with community infections, can cause neutrophil lysis after phagocytosis by neutrophils. Treatment with antitoxin antibodies to prevent neutrophil lysis may be an effective therapeutic strategy [80]. A lack of neutrophils after bacterial infection can lead to severe infections and ulcers [81]. In the presence of *S. aureus* biofilms on the implant surface, the expression of receptors for neutrophil recognition and bactericidal activity at the infection site is upregulated, while the expression of factors required for neutrophil migration is downregulated. Therefore, neutrophils will continue to exist on the surface of the biofilms but will not migrate into the biofilms to remove the biofilms, and persistent infection will cause tissue damage [51].

### Myeloid-derived suppressor cells (MDSCs)

MDSCs are a heterogeneous population of cells, including bone marrow progenitor cells, immature macrophages, immature granulocytes, and immature dendritic cells (DCs). They are a unique component of the immune system and have a remarkable ability to inhibit the T-cell response. They are immature in the bone marrow and amplified during infection, inflammation, and cancer [82, 83]. MDSCs can be classified as granulocyte or polymorphonuclear MDSCs (PMN-MDSCs) and monocyte MDSCs (M-MDSCs). Studies on humans have shown that MDSCs also contain cells with colony-forming activity and other myeloid precursor cells [84].

PMN-MDSCs and M-MDSCs have different immunosuppressive mechanisms. Factors related to MDSC activity include the upregulation of ARG1, NO, and ROS and the production of prostaglandin E2 (PGE2). The ER stress response is a mechanism that has emerged in recent years to regulate MDSCs. There are three types of ER stress response sensors: protein kinase RNA-like ER kinase (PERK), inositol-requiring enzyme 1 (IRE1), and activating transcription factor 6 (ATF6). Condamine and Thomas et al. found that MDSCs from tumor-bearing mice and patients with cancer demonstrate a much greater ER stress response than neutrophils and monocytes from tumor-free hosts [84, 85].

Many studies have shown that bacteria can induce and regulate MDSCs in vivo and in vitro. The number of MDSCs that inhibit T cells increases in *S. aureus* and *Mycobacterium tuberculosis* infections and in patients with sepsis. However, the increase in MDSCs after bacterial infection does not always show a negative effect on the host. Poe et al. found that in *Pseudomonas aeruginosa* and *Klebsiella pneumoniae* infections, increased MDSCs are associated with host protection and better outcomes. In the early stages of bacterial infection, neutrophils and macrophages protect the host when MDSCs are absent or in small numbers. However, as the infection persists, MDSCs increase and suppress adaptive immunity [84, 86]. MDSCs may be responsible for the persistence of bacterial biofilms that cannot be removed [87]. Heim et al. found that IL-12 plays an important role in recruiting MDSCs to the biofilm formation site to promote persistent bacterial infection [88].

### Other cells

There is increasing evidence from infected animal models that bacterial infection activates polymorphonuclear neutrophils and T cells and proliferates them. Activation of T cells has also been observed in patients with multiple bacterial infections. In patients with implant-associated osteomyelitis, the number of T cells was second only to that of polymorphonuclear neutrophils. CD4 + and CD8 + T cells were detected in the peripheral blood of the patients, and CD8 + T cells were predominant. Most of these T cells are terminally differentiated effector T cells [89–93].

At the infection site, polymorphonuclear neutrophils and T cells regulate each other. A variety of neutrophil-derived cytokines regulate T cell differentiation. For example, IL-4 and IL-12 derived from polymorphonuclear neutrophils play a key role in regulating the functional differentiation of T cells. T cell-derived cytokines such as IL-2 and IFN-γ also activate and prolong the life span of polymorphonuclear neutrophils [89, 94–98].

Mast cells produce cytokines in the inflammatory response that promote the recruitment of leukocytes and monocytes [71]. At present, the specific mechanism of DCs in the immune response is not clear, but relevant studies have found that DCs can act as messengers between innate and adaptive immunity, so implants can also regulate mast cells and DCs to achieve immune regulation [99–101].

## Novel immune-enhanced antimicrobial strategies

**Fig. 3:**
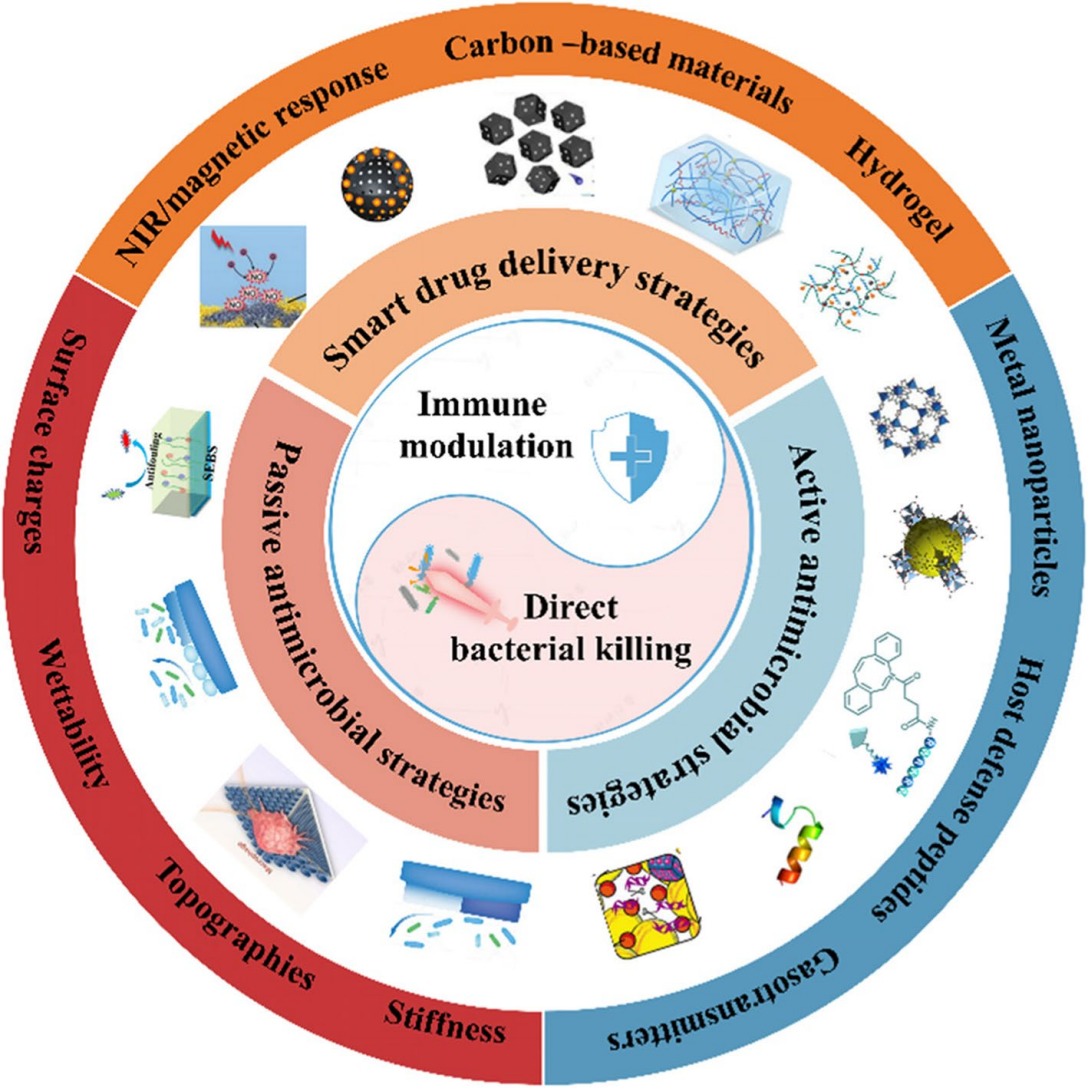
Schematic illustration of novel immune-enhanced antimicrobial strategies. Left: Passive immune-enhanced antimicrobial strategies work by surface modification of biomaterials such as surface morphology, wettability, stiffness and surface charge. Right: Active antimicrobial strategies are achieved by loading biomaterials with various bioactive molecules such as metal nanoparticles, host defense peptides and donators of gasotransmitters. Up: Smart drug delivery strategies for the responsive release of drugs through carbon-based materials and hydrogels. Reproduced with permission [102–111]. Copyright 2021, American Association for the Advancement of Science; Copyright 2019, American Chemical Society; Copyright 2021, American Chemical Society; Copyright 2022, American Association for the Advancement of Science; Copyright 2021, Wiley-VCH GmbH; Copyright 2020, American Chemical Society; Copyright 2015, Royal Society of Chemistry; Copyright 2021, Elsevier Ltd; Copyright 2020, American Chemical Society; Copyright 2021, Elsevier B.V.

Previous designs for antimicrobial materials often failed to eradicate infections because they focused on the direct bactericidal ability of the biomaterials and neglected the regulation of immune cells. The relationship between the direct bactericidal ability and immunomodulatory effect of biomaterials is analogous to the concept of “yin and yang” in Chinese culture, which is mutually reinforced and complementary in combating IAI (Fig. 3). Novel immune-enhanced antimicrobial strategies for implants can be classified as active and passive. Passive antimicrobial strategies inhibit bacterial function or modulate immune activity by surface modification of biomaterials such as surface morphology, wettability, stiffness, and surface charge. Active antimicrobial strategies are achieved by loading biomaterials with various bioactive molecules such as metal nanoparticles and host-defense peptides. The two antimicrobial strategies are not completely separate. It is usually difficult to classify some of these new technologies, as most are still in the early stages of development, and some belong to both groups. Although the two antibacterial strategies are slightly different, they both emphasize the synergy of direct bacteria-killing and immune modulation.

### Passive immune-enhanced antimicrobial strategies

**Fig. 4:**
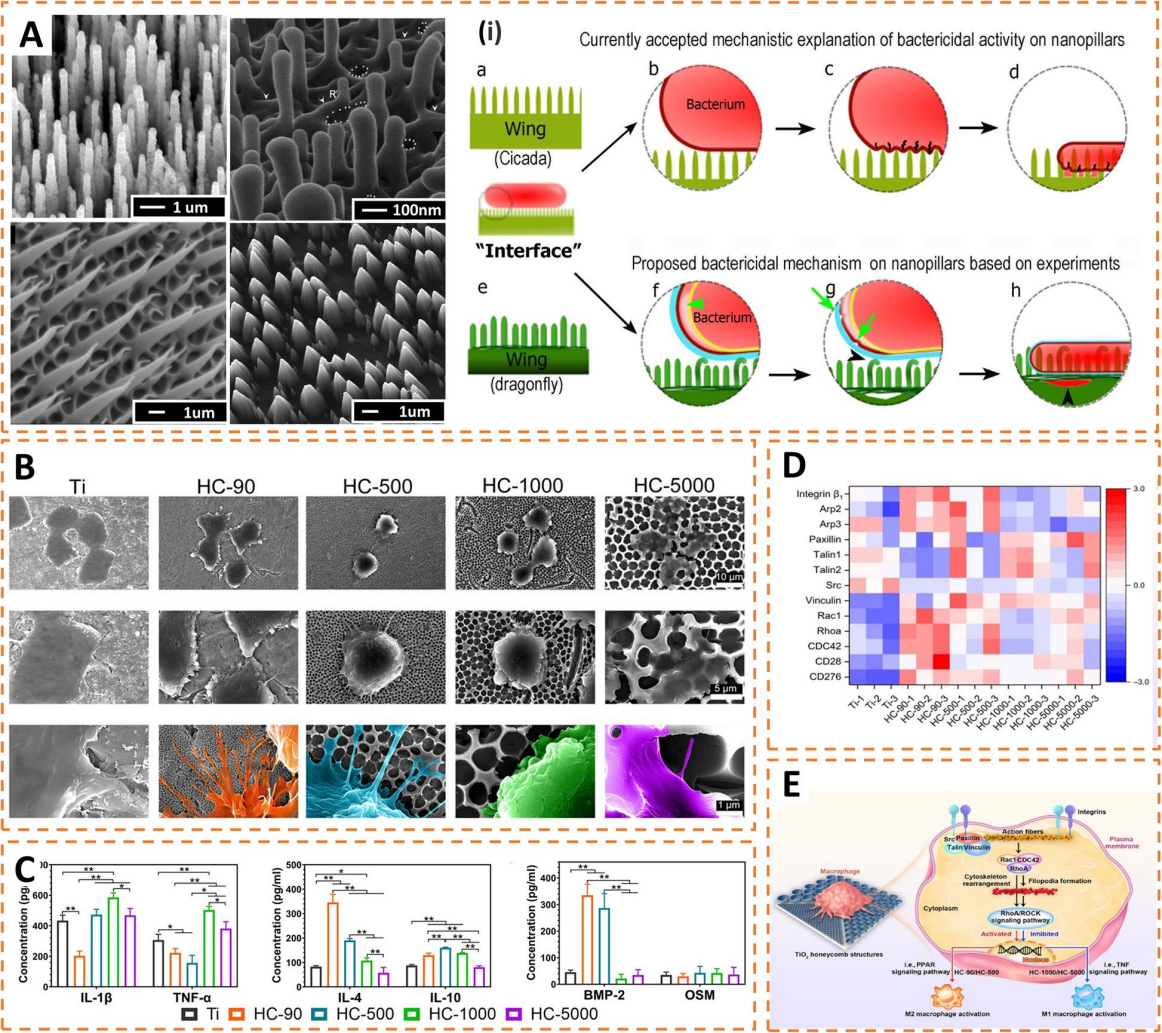
Influence of biomaterial surface topography on the behavior of bacteria and immune cells. **A** SEM images of antibacterial topographical features of animal skins and corresponding biomimetic nano-structured surface (biomimetic needles, dragonfly wing, gecko skin and biomimetic diamond nanocone surfaces). (i) Proposed bactericidal mechanisms of the nanopillars on the surface of dragonfly wings and cicada wings. Green arrows indicate separation of bacterial membrane structures. Reproduced with permission [114–117]. Copyright 2018, Royal Society of Chemistry; Copyright 2017, American Chemical Society; Copyright 2015, Royal Society; Copyright 2016, American Vacuum Society. **B** Corresponding morphological changes of RAW 264.7 cells on different sizes of honeycomb–like TiO2 nanostructures. **C** ELISA analyses of cytokines secreted by macrophages attached to different TiO2 structures (IL-1β, TNF-α, IL-4, IL-10, BMP-2 and OSM). **D** Heatmap analysis of differentially expressed genes related to cytoskeleton arrangement, cell adhesion and mechanotransduction. **E** Schematic illustrating the mechanism by which surface topography affects macrophage polarization. Reproduced with permission [102]. Copyright 2021, American Association for the Advancement of Science

Passive immune-enhanced antimicrobial strategies enhance intrinsic antimicrobial activity by changing the chemical properties and topology of the biomaterial surface. Above we have described the adhesion process of bacteria to the surface of implants. The surface’s chemical properties (e.g., hydrophobicity and surface charges) and topology of biomaterials can significantly affect the interactions between the material’s surface and the bacteria [112]. Therefore, the strategy of inhibiting bacterial adhesion or even killing bacteria directly by altering the surface characteristics of implants is reasonable and is believed to slow down the development of bacterial resistance. Changing the nanotopography of the material is the simplest method, as inspired by the patterns on the surfaces of plants and animals with antimicrobial activity (e.g., cicadas, dragonflies, and lotus leaves) (Fig. 4 A) [113–117]. The bactericidal mechanism of nanostructures on material surfaces is unknown and may involve overstretching and rupture of bacterial cell membranes on material surfaces. However, the morphology, size, spacing, and sharpness of nanostructures all affect bactericidal efficiency. Furthermore, nanostructures tend to be more effective in bactericidal activity against Gram-negative bacteria. Compared to Gram-positive bacteria, Gram-negative bacteria have fewer peptidoglycan layers (1–3 layers) in their walls and lower maximum membrane stretching capacity, leading to increased cell death. Due to the influence of multiple factors on the antibacterial efficiency, no specific micropattern has been found so far to kill all types of microorganisms. It remains challenging to design a universal nanopattern to resist multiple bacteria.

Surface morphology also has a significant impact on the behavior of immune cells. In this regard, macrophages are the most widely studied cells. Macrophages are sensitive to changes in material properties. Several studies have reported the modulation of macrophage phenotype and function via the surface topography of biomaterials. For example, Zhu et al. found that changing the diameter of TiO_2_ nanotubes could regulate macrophage polarization and cytokine secretion (Fig. 4B, C). Decreasing the diameter of TiO_2_ nanotubes significantly activated the anti-inflammatory M2 macrophages and promoted the expression of anti-inflammatory genes. Transcriptomic analysis revealed that the tube structure with smaller diameters promoted the formation of filopodia and upregulated integrins, ARP, and the Rho family of GTPases (Rac1, RhoA, and Cdc42) (Fig. 4D, E), which tends to promote macrophage polarization toward the M2 phenotype [102]. Similarly, Chen et al. investigated topography-induced behavior changes in macrophages through parallel gratings (line width 250 nm-2 μm) imprinted on poly(lactic acid) (PLA), poly(epsilon-caprolactone) (PCL), and poly(dimethyl siloxane) (PDMS). Compared to controls, the maximal adhesion and elongation of macrophages were found on 500 nm gratings over 48 h. Furthermore, TNF-α and VEGF levels secreted by macrophages also showed sensitivity to topography, with reduced levels observed at larger grating sizes [118].

Although neutrophils are important mediators of the initial inflammatory response to infection, limited studies explored the effect of surface structure on neutrophils. Zhang et al. observed that micromorphic structures induced neutrophil death and ROS production [11]. In addition, some studies have observed that increased hydrophobicity and elasticity of the material also promote the release of pro-inflammatory factors from neutrophils and the formation of NETs [119, 120]. The role of neutrophils in the immune response to biomaterials is not fully understood. More research is needed in the future to elucidate the functions of neutrophils in regulating the biomaterial interfaces in the fight against infection.

### Active immune-enhanced antimicrobial strategies

To further enhance the antimicrobial activity of the biomaterial, additional molecular and ionic functionalization can be performed. A variety of compounds such as metal nanoparticles and host defense peptides have shown antibacterial and immunomodulatory activities. The loading of such compounds allows implants to switch from passive to active antimicrobial. In this chapter, we discuss the promising applications of these materials in detail.

#### Metal nanoparticles

Metal nanoparticles have a long history as antimicrobial agents and have a wide antibacterial spectrum. Their antibacterial mechanisms include damage to cell walls, oxidative stress induction, and bacterial metabolism inhibition [121]. Coupling metal ions to the surface of biological materials is an economical and reliable method. Recent studies have found that metal particles can trigger an effective anti-infective response in vivo even at concentrations below the minimum inhibitory concentration [122, 123]. It is indicated that metal ions exert their antibacterial functions through direct antibacterial activity and by regulating the immune response. Table 1 summarized the biological functions of some metals and their advantages and limitations as immunotherapeutic agents. In the following section, the antimicrobial and immunomodulatory effects of silver (Ag), gallium (Ga), and zinc (Zn) in the design of anti-infective biomaterials are discussed in detail.

**Table 1:** Overview of metals with respect to their antibacterial effects, immunomodulatory effects, advantages and limitations

	Antibacterial effects	Immunomodulatory effects	Advantages	Limitations	Ref
Ag	· Disrupt membranes by binding to sulfhydryl groups · Damage DNA and protein by oxidative stress · Bind directly to nucleic acid molecules and hinder cell growth and reproduction	· Induce M1 polarization of macrophages and exacerbate the inflammatory response · Promote M2 polarization of macrophages, ROS scavenging, and new bone formation (in very low doses) · Induce death of host cells	· Direct and broad-range antibacterial effects · High stability · Mature techniques available for incorporation in implants	· Toxicity for eukaryotic cells	[124–126, 135, 136]
Ga	· Replace iron in bacterial metabolism · Aggregate at the site of inflammation	· Inhibit bone resorption by inhibiting osteoclast activity · Promote osteogenic gene expression in MG63 cells · Reduce the inflammatory response of the wound	· Unique antibacterial mechanism · Inhibit bone resorption · Aggregate spontaneously to the site of infection	· Unstable antibacterial effect influenced by iron concentration · Fewer molecular mechanism studies currently · High cost	[140, 141] [143, 144, 195]
Zn	· Damage DNA and protein by oxidative stress · Disrupt the cell walls/membranes	· Enhance phagocytosis and chemotaxis of macrophages · Mediate PGRPs exert antibacterial activity · Promote immune cell maturation · Anti-inflammatory effects by deregulating NF-κB pathway activity	· Pro-osteogenic effects described · Significant immune regulation function · Essential trace elements	· Weak direct antibacterial effects	[149–151, 154–156] [157, 158]
Cu	· Disrupt membranes by binding to sulfhydryl groups Generate ROS through Fenton reactions · Disrupt helix structure of DNA	· Facilitate M1 macrophage switch and bacteria phagocytosis to resist infection · Induce a proper immune environment for bone regeneration · Induce angiogenesis	· Direct and broad-range antibacterial effects · Fenton reaction catalyst · High stability · Essential trace elements · Significant angiogenic and osteogenic abilities	· Toxicity at supraphysiologic concentration	[159, 196, 197]
Mg	· Inhibit bacterial adhesion and biofilm formation due to Elevated pH by Mg degradation · Inhibit biofilm-forming genes and protein expression in the outer membrane of bacteria	· Stimulate M2 macrophage switch and inhibit osteoclastogenesis and osteolysis · Downregulate pro-inflammatory cytokines and induce osseointegration · Protect neutrophils from endogenous oxidative stress	· Essential element for bone tissue · Excellent anti-inflammatory and pro-osteogenic activity	· Poor corrosion resistance · Poor inherent antibacterial properties	[198–200]

**Fig. 5:**
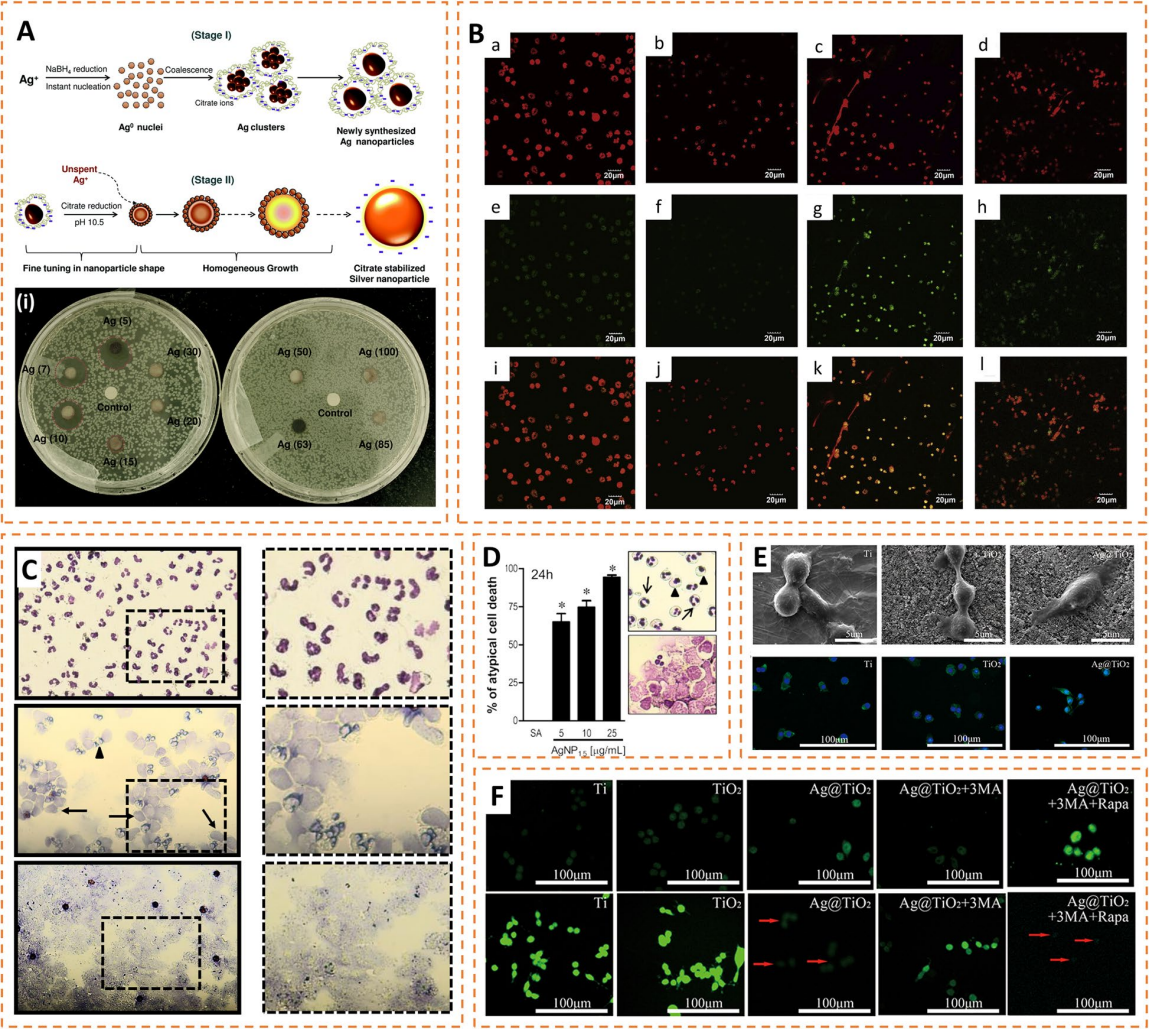
Antibacterial and immunomodulatory effects of AgNPs. **A** Schematic illustration indicating the synthesis of different sized AgNPs by co-reduction approach. (i) Comparison of the antibacterial effect against *E. coli* of different sizes of AgNPs by inhibition zone experiments. Reproduced with permission. Copyright 2014, Royal Society of Chemistry. **B** Fluorescence images visualizing the formation of NETs induced by AgNPs. Reproduced with permission [132]. Copyright 2019, Elsevier Inc. **C** Optical microscope images showing AgNP-induced atypical cell death of PMNs distinct from necrosis. (Up: freshly isolated PMNs; middle: AgNP-induced atypical cell death; down: heat-induced necrosis). **D** Percentage of atypical cell death of PMNs induced by AgNPs at different incubation concentrations for 24 h (0, 10 and 25 µg/mL). The insets show the atypical death of neutrophils on the optical microscope. Reproduced with permission [134]. Copyright 2015, Elsevier B.V. E SEM images and fluorescent images showing the morphology of macrophages cultured on different surfaces (Left: Ti; Middle: TiO_2_-NTs; Right: Ag@TiO_2_-NTs). F Fluorescence images and analysis of ROS and LC3 in macrophages grown on different samples (Ti, TiO_2_-NTs, Ag@TiO_2_-NTs, Ag@TiO_2_-NTs + 3MA and Ag@TiO_2_-NTs + 3MA + Rapa). Reproduced with permission^118^. Copyright 2020, Dove Press Ltd

Ag nanoparticles. Silver is a classic and widely used antibacterial agent whose complex antibacterial mechanisms have not been fully revealed. Several excellent reviews have investigated the potential antibacterial mechanisms of silver, involving disruption of bacterial membrane structure, induction of excess ROS, and destruction of proteins and nucleic acids [124–126]. Significantly, the bactericidal effect of AgNPs also depends on their properties (e.g., size, shape, surface charge, and particle dispersion state) [127]. Specifically, smaller size nanoparticles (less than 10 nm) were found to possess stronger antibacterial activity against *E. coli* (Fig. 5 A), which may result from the fact that the small-sized nanoparticles are more likely to adhere to and penetrate the bacterial membrane structure [128].

Despite the strong antibacterial activity, the toxicity of Ag to mammalian cells largely limits its application in prosthetic coatings. In vivo, Ag has been reported to cause inflammatory responses in various organs and tissues, such as the lung, liver, heart, and brain [129–131]. Wang et al. reported that silver nanoparticles induced NETs release significantly, and the potential mechanisms were related to the production of reactive oxygen species (ROS) depending on NADPH oxidase and MAPK signaling pathways (Fig. 5B) [132]. Similar results were observed by Kang et al. [133]. Liz et al. reported that silver nanoparticles (AgNPs) rapidly induce atypical cell death of neutrophils within 60 min, which involves ROS production and inflammatory caspase-1 and caspase-4 (Fig. 5 C, D) [134]. For macrophages, some studies suggest that silver ions exacerbate the inflammatory response by inducing M1 polarization in macrophages, which is mediated by ROS and the NF-kB pathway [135, 136]. The local inflammatory environment promotes the formation of osteoclasts, which is detrimental to osteogenesis. In addition, Zielinska et al. found that AgNPs also elevated the production of NO and its derived reactive molecules, leading to osteoblast death by inducing increased expression of iNOS [137]. Interestingly, several studies reported that very low concentrations of silver ions inhibited the inflammatory process and promoted bone healing by inducing M2 polarization, which involved the scavenging of ROS and enhanced autophagy (Fig. 5E, F) [138, 139]. The immune effects of silver are so sophisticated that further studies are still needed to elucidate the mechanisms. In conclusion, silver still has great potential as an alternative antibacterial agent to antibiotics. In the future, it is hoped that the toxicity of silver can be reduced with more efficient methods so that silver ions will have greater applications.

Ga nanoparticles. Gallium compounds have long been used as anticancer drugs. Recently, the anti-infective effect of gallium has been gradually discovered [140, 141]. The antibacterial activity of gallium originates from its chemical similarity to iron. The bacterial biological system cannot distinguish gallium from iron, so gallium can be effectively absorbed and participate in the biochemical process of bacteria. The reduction reaction of Fe^3+^ ions is a key step in the metabolism of bacteria in cellular respiration, oxygen transport, and DNA synthesis. Unlike Fe^3+^, Ga^2+^ cannot be reduced under physiological conditions, thus hindering multiple metabolic processes in bacteria and resulting in bacterial death. Moreover, Ga^3+^ is enriched with proteins such as transferrin and lactoferrin due to the more severe inflammation at the site of infection, which increases the local effective drug concentration.

Previously, gallium was used to treat hypercalcemia of malignant tumors [142]. Gallium can inhibit bone resorption by suppressing osteoclast activity without interfering with osteoblast activity, which raises expectations for its application in orthopedics. Bonifacio et al. constructed a gallium-chitosan coating on titanium plants by electrochemical deposition [143]. Compared with the titanium tablet group, the gallium coating promoted the expression of osteogenic genes in MG63 cells.

Some studies have reported the anti-inflammatory effects of gallium. For example, Dong et al. developed gallium-doped TiO_2_ nanotube coatings (TNTs) on the surface of gallium. In a murine infection model, rats implanted with gallium-doped TNTs after operation showed mild inflammation and a minimal surgical scar area [144]. Similarly, in another wound model infected with *K. pneumoniae*, gallium citrate (GaCi) treatment resulted in faster skin wound closure with reduced inflammation [145]. The anti-inflammatory effect of gallium may be related to its inhibition of leukocyte inflammatory factors such as IL-1β, IL-6, and TNF-α, the reduction of oxidative stress, and interference with matrix metalloproteinase (MMP) activity, and antagonism of the proinflammatory effect of iron. As a new antibacterial agent, the precise immunomodulatory function of gallium needs to be further explored.

**Fig. 6:**
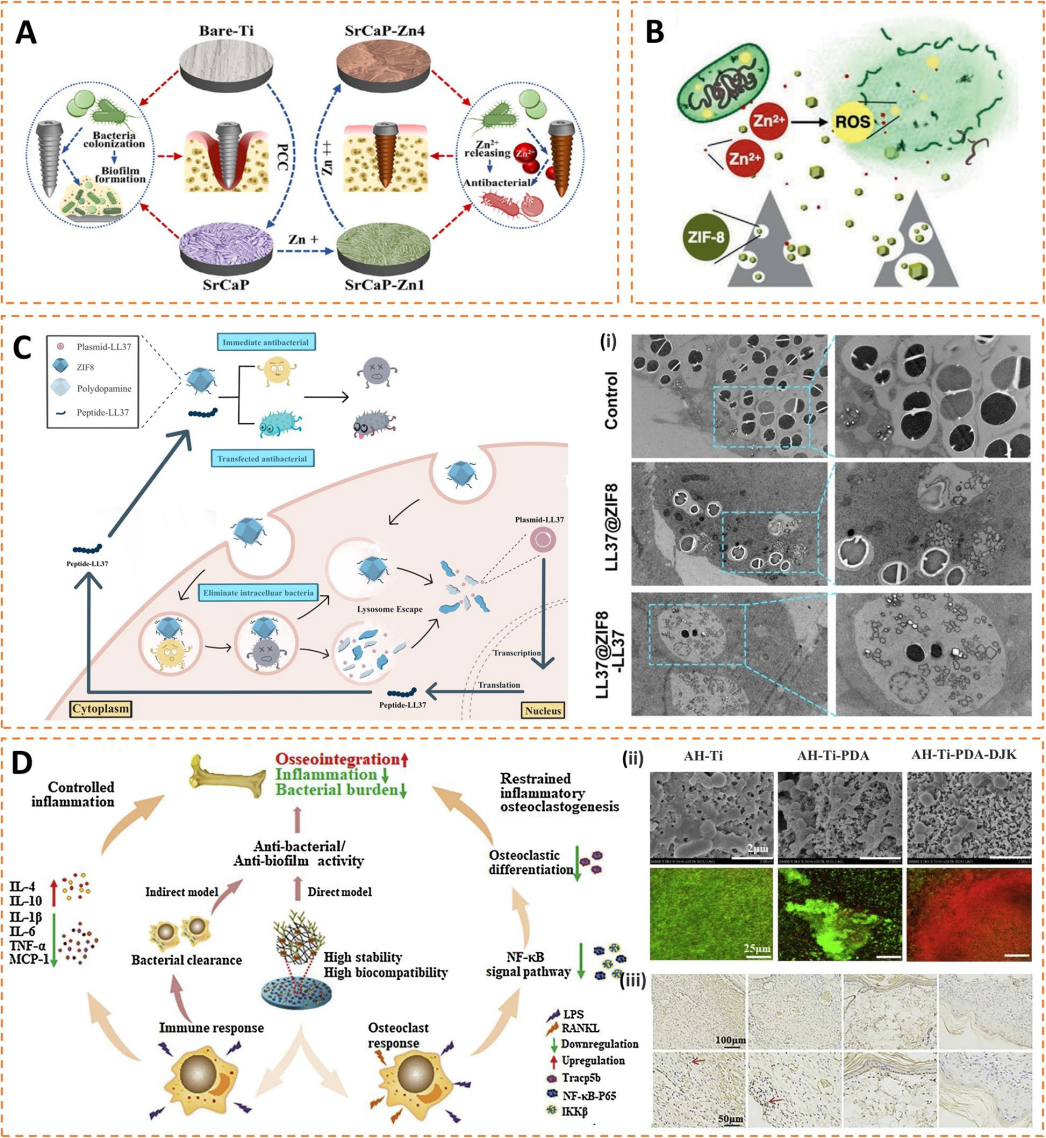
**A** Schematic image illustrating the functionalization of the orthopedic implants with Zn and its antibacterial mechanism to prevent IAI. Reproduced with permission [152]. Copyright 2022, American Chemical Society. **B** Schematic illustration of the antibacterial mechanism of Zn-MOFs involved in ROS production [153]. Copyright 2021, Wiley-VCH GmbH. **C** Schematic diagram of the comprehensive antibacterial strategy based on LL37, including bacteria killing directly and producing LL37 continuously against escaped bacteria. (i) TEM images illustrating the ability of LL37 to kill intracellular bacteria (Up: control; middle: LL37@ZIF8; down: LL37@ZIF8-LL37). Reproduced with permission [182]. Copyright 2022, Wiley-VCH GmbH. **D** Schematic illustration of DJK-5-decorated surface with antimicrobial, immunomodulatory and osteolysis-inhibiting properties. (ii) SEM images and Live/dead staining showing the destruction of multiple bacterial adhered on HDP-functionalized surfaces (AH-Ti, AH-Ti-PDA and AH-Ti-PDA-DJK-5, respectively). (iii) Immunohistochemical (IL-6) staining demonstrating the anti-inflammatory ability of the DJK-5 immobilized surface (Gauze, AH-Ti, AH-Ti-PDA and AH-Ti-PDA-DJK-5, respectively). Reproduced with permission [183]. Copyright 2021, Elsevier B.V.

Zn nanoparticles. As an important trace element, zinc is not only a component of most proteins and enzymes but also widely involved in the metabolism of nucleic acids, sugars, and lipids and the regulation of gene transcription and other important processes [146–148]. Therefore, zinc plays a critical role in growth, development, heredity, and immunity. Some studies have reported the inhibitory effect of zinc oxide on Gram-negative and Gram-positive bacteria (Fig. 6 A) [149–153]. Its antibacterial mechanism also involves damage to bacterial cell walls and oxidative stress (Fig. 6B), similar to silver.

Besides, the immunoregulatory role of zinc may be even more important. Zinc deficiency can cause immaturity of immune cells and accelerate apoptosis of pre-B and pre-T cells, thereby reducing cell resistance to pathogens [154]. Zinc is also related to the anti-infective ability of various innate immune cells [148]. With zinc deficiency, the chemotactic and phagocytic activities of the PMN are reduced. The recognition of class I major histocompatibility complex (MHC) by NK cells and the lytic activity of NK cells are also affected by zinc consumption. For macrophages, circulating monocytes must be attracted to the target tissue and adhere to endothelial cells before they mature into tissue-resident cells. Zinc enhances this adhesion process.

In addition, zinc can exert its anti-inflammatory effect by negatively regulating the NF-κB signaling pathway, which is related to the increase in zinc-dependent zinc finger protein A20 and the expression of peroxisome proliferator-activated receptor α (PPAR-α) [155, 156]. Zinc deficiency increases the production of proinflammatory cytokines, such as interleukin IL-1β, IL-6, and tumor necrosis factor (TNF) α, which may exacerbate damage to normal tissue. Another immunomodulatory function of zinc is related to peptidoglycan recognition proteins (PGRPs) [157, 158]. PGRPs are a class of highly conserved pattern recognition receptors essential for recognizing peptidoglycan structures in bacteria. Wang et al. found that PGRPs depend on zinc to exert their antibacterial activity [158].

Other metal nanoparticles. The biological activity of other metal nanoparticles is also of interest. For example, copper (Cu) is being tried to produce implant antimicrobial coatings because of its great antibacterial properties. Cu^2+^ can not only damage the membrane structure of bacteria but also produce a large amount of toxic hydroxyl radicals (•OH) to kill bacteria by facilitating a Fenton-like reaction [159]. Cu is also an essential micronutrient for maintaining optimal innate immune function. In vivo, copper deficiency leads to increased susceptibility to bacterial infection associated with compromised activity and number of neutrophils and macrophages [160]. Huang et al. observed that Cu-doped titanium implants enhanced the bactericidal effect of macrophages by promoting their M1 polarization [123]. Interestingly, this immunomodulatory activity was observed at a Cu^2+^ concentration of 0.4 ppm, far lower than its minimum inhibitory concentration. Moreover, copper-doped biomaterials exhibit significant angiogenic and osteogenic abilities in many studies [159]. As a promising immunomodulatory material, more research is needed in the future to clearly understand how copper ions interact with the physiological processes of immune cells and even bone cells to guide clinical applications.

Magnesium (Mg) is another metal that has received considerable attention. More than 50% of magnesium in the human body is stored in bone tissue [161]. Therefore, Mg is vital for the maintenance of normal bone health. Magnesium-deficient animals have reduced numbers of osteoblasts and reduced bone mass, leading to osteoporosis [162]. Mg is considered an ideal orthopedic implant material because of its gradual degradation properties, avoiding implant removal surgery and its associated complications (such as IAI). Most studies have reported the excellent anti-inflammatory activity of Mg, which facilitates the osseointegration of implants. For example, Qiao et al. fabricated a Mg^2+^-doped titanium dioxide nanotube coating on titanium surfaces by anodic oxidation and hydrothermal treatment to understand the interaction of osteoblasts and immune cells with the material [163]. The Mg^2+^-doped titanium surfaces polarized macrophages toward the anti-inflammatory M2 phenotype and promoted the expression of osteogenic-related proteins (VEGF and BMP2). In vivo, Mg^2+^-doped implants induced more anti-inflammatory macrophages and promoted more blood vessel and bone trabecula formation than bare Ti implants. However, Mg has limited antimicrobial properties [164]. Future research will focus on how to increase the inherent antimicrobial capacity of Mg and achieve controlled degradation.

#### Host defense peptides

Host defense peptides (HDPs), as naturally occurring antibiotics in the body, have broad-spectrum antibacterial activity. Some HDPs also show a killing effect on fungi, viruses, and cancer cells [165–167]. Most HDPs are composed of 12–50 amino acid residues that are rich in basic amino acids (Arg and Lys) and hydrophobic amino acids (Leu, Ile, Phe, Val, and Trp). Therefore, host defense peptides exhibit strong cationic and amphiphilic characteristics in physiological environments, which underlies their antibacterial activity [168]. The positive charge ensures the aggregation of polyanionic microorganisms on the cell surface. Afterward, HDPs destroy the integrity of the membrane or transfer into the cell to interfere with DNA replication, transcription, and other biological processes. Some studies found that HDPs showed anti-biofilm effects at concentrations lower than those required to kill planktonic cells [168, 169]. It is suggested that the anti-biofilm effect of AMP may follow a different mechanism from that of anti-planktonic bacteria, such as inhibition of bacterial adhesion, inhibition of biofilm maturation, and inhibition of bacterial stringent response.

It has been observed that the direct bactericidal effect of many antimicrobial peptides is severely diminished under physiological conditions (such as in physiological salt solutions and plasma) [170, 171]. Some HDPs with little antimicrobial efficacy in vitro exhibit an active ability to control infection in vivo [172, 173]. It has been speculated that HDPs may rely more on mobilizing immune cells to fight bacteria, and some studies confirmed this idea. For instance, Scott et al. reported an immunomodulatory peptide (IDR-1) that exerts antibacterial activity against a variety of bacteria, including MRSA, in mouse models by regulating the response of monocytes and macrophages rather than relying on its direct antibacterial activity [174].

The immunomodulatory function of HDP goes far beyond that. Yang et al. found that the human cathelicidin-like antimicrobial peptide LL-37 can attract multiple immune cells, such as neutrophils, monocytes, and T cells, via formyl peptide receptor-like 1 (FPRL1) [175]. Does et al. indicated that LL-37 induced monocytes to differentiate into the M1 pro-inflammatory phenotype during the differentiation of monocytes into macrophages [176]. Herster et al. reported that LL-37 is associated with the formation of NETs and the self-amplifying inflammation [177]. Interestingly, some studies also reported the anti-inflammatory effects of this HDP. Mookherjee et al. found that LL-37 stimulation can lead to higher levels of anti-inflammatory IL-10 in pDCs, myeloid DCs (MDCs), monocytes, B cells, and T cells than in untreated cells [178]. Other HDPs, such as human defensins, have also been found to have both pro- and anti-inflammatory effects. In addition, host defense peptides also activate antigen-presenting cells, promote DC maturation, inhibit tumor growth, promote angiogenesis and wound healing, enhance intestinal homeostasis, induce apoptosis of some cells, etc. Table 2 summarized the source and biological functions of HDPs as well as their advantages and limitations as immunotherapeutic agents in detail. The immunomodulatory activity of HDP is broad and complex. A slight perturbation in the immune response may seriously affect the organism, producing unpredictable off-target effects. Therefore, the functions of HDP and its interconnections with other immunologically active substances should be systematically considered when developing HDP-based immunomodulatory therapies.

**Table 2:** Overview of HDPs with respect to their expression patterns, biological functions, advantages and limitations

	HDP‑expressing cells	Antibacterial effects	Immunomodulatory effects	Advantages	Limitations	Ref
LL-37	· Macrophages · Neutrophils · NK cells · Mast cells · Monocytes · lymphocytes · Epithelial cells	· Disrupt bacterial walls/membranes (The models of membrane disruption proposed include aggregate, toroidal, barrel-stave, and carpet models) · Inhibit internal targets (such as protein synthesis, DNA/RNA synthesis, translation, and protein folding)	· Recruit immunocytes (such as neutrophils, eosinophils, monocytes, and T cells) · Induce apoptosis in some cell types (such as epithelial cells and regulatory T cells) and inhibit neutrophil apoptosis · Induce Mast cell degranulation to enhance diapedesis · Inhibit proinflammatory responses selectively · Induce M1 macrophage switch and upregulate phagocytosis · Facilitate angiogenesis and wound healing · Induce cytokine and chemokine production (such as CXCL8, and CCL7) · Adjuvant effects	· Powerful and broad-range immunomodulator functions · Inflammation suppressive effects · Prohealing effects · Synergistic interaction with antibacterial agents · Pro-osteogenic effects described · Anti-biofilm/Antifungal/Antiviral activity	· Weak direct antibacterial effects · Limited preclinical evidence for IAI prevention · High cost of production · Limited stability in vivo · Limited tissue penetration	[165–167] [175–177]
Defensins	· Macrophages · Neutrophils · NK cells · Mast cells · Monocytes · DCs	· Disrupt bacterial walls/membranes (The models of membrane disruption proposed include aggregate, toroidal, barrel-stave, and carpet models) · Inhibit internal targets (such as protein synthesis, DNA/RNA synthesis, translation, and protein folding)	· Recruit immunocytes (such as neutrophils, eosinophils, monocytes, and T cells) · Induce cytokine and chemokine production (such as CXCL8, IL-6, CCL2, and GM-CSF) · Induce apoptosis in some cell types and inhibit neutrophil apoptosis · Promote or inhibit inflammatory · Promote or inhibit angiogenesis; promote wound healing · Maintain gut homeostasis · Form nanonets · Adjuvant effects	· Powerful and broad-range immunomodulator functions · Prohealing/angiogenic effects · Synergistic interaction with antibacterial agents · Antifungal/Antiviral activity · Each type of defensins has a unique role in innate immunity	· Weak direct antibacterial effects · Induce undesirable proinflammatory processes around the biomaterial. · Unpredictable off-target effects · Limited preclinical evidence for IAI prevention · High cost of production · Limited stability in vivo · Limited tissue penetration	[165–168]

The application of HDP to biomaterials has attracted widespread interest. Unlike conventional antibiotics with a single antibacterial mechanism, HDPs have multiple regulatory targets, making it difficult for bacteria to develop resistance [179–181]. For instance, Qu et al. developed a synergetic active antimicrobial and immunotherapeutic strategy based on HDPs (Fig. 6 C). They used a porous nanomaterial (ZIF8) to encapsulate the LL-37 plasmid and grafted LL-37 onto the material surface. LL-37 grafted on the material surface killed bacteria inside and outside the cells. Furthermore, after translation of the plasmid, the host cells not only sustained the production of LL-37 but also significantly suppressed the abnormal increase of MDSCs in the local infected microenvironment, thereby alleviating the immunosuppressed state and restoring the protective immune response against bacteria [182]. Wang et al. introduced DJK-5 (an HDP based on IDR-1018) into porous titanium alloys to create DJK-5 functionalized surfaces. DJK-5 imparts a variety of functional properties to immobilized surfaces, including antimicrobial ability, immunomodulation, osteolysis inhibitory properties, and biocompatibility. DJK-5 not only directly disrupted the integrity of bacterial membranes to kill bacteria but also enhanced the bactericidal properties of macrophages. The results showed that the antibacterial rate of the DJK-5 functionalized surface exceeded 90% for both Gram-positive and Gram-negative bacteria. In addition, DJK-5 functionalized surface could modulate the immune response to suppress the excessive inflammatory response induced by severe infections, thereby inhibiting osteoclast differentiation induced by the inflammatory environment (Fig. 6D) [183].

To date, most clinical trials have focused on the local application of HDPs to address surface infections, implying concerns about the toxicity of HDPs [184, 185]. Some natural host defense peptides, such as LL-37 and defensin, have a narrow difference between antimicrobial and toxic concentrations, which greatly limits their application [186, 187]. Furthermore, the sensitivity to protease and high production cost also hinder its application. A series of strategies have been proposed to address these problems, including replacing L-amino acids (L-AAs) with D-amino acids (D-AAs), combining HDP with polymers or macromolecules, and developing peptide mimics with similar structures and activities [188–190]. Various new HDP carriers, including delivery carriers such as polymers and lipid nanoparticles, are also being developed to improve the stability and biocompatibility of HDPs. The growing worldwide interest in exploring the functions and applications of HDPs reflects the great potential of HDPs for future use.

#### Gasotransmitters

**Fig. 7:**
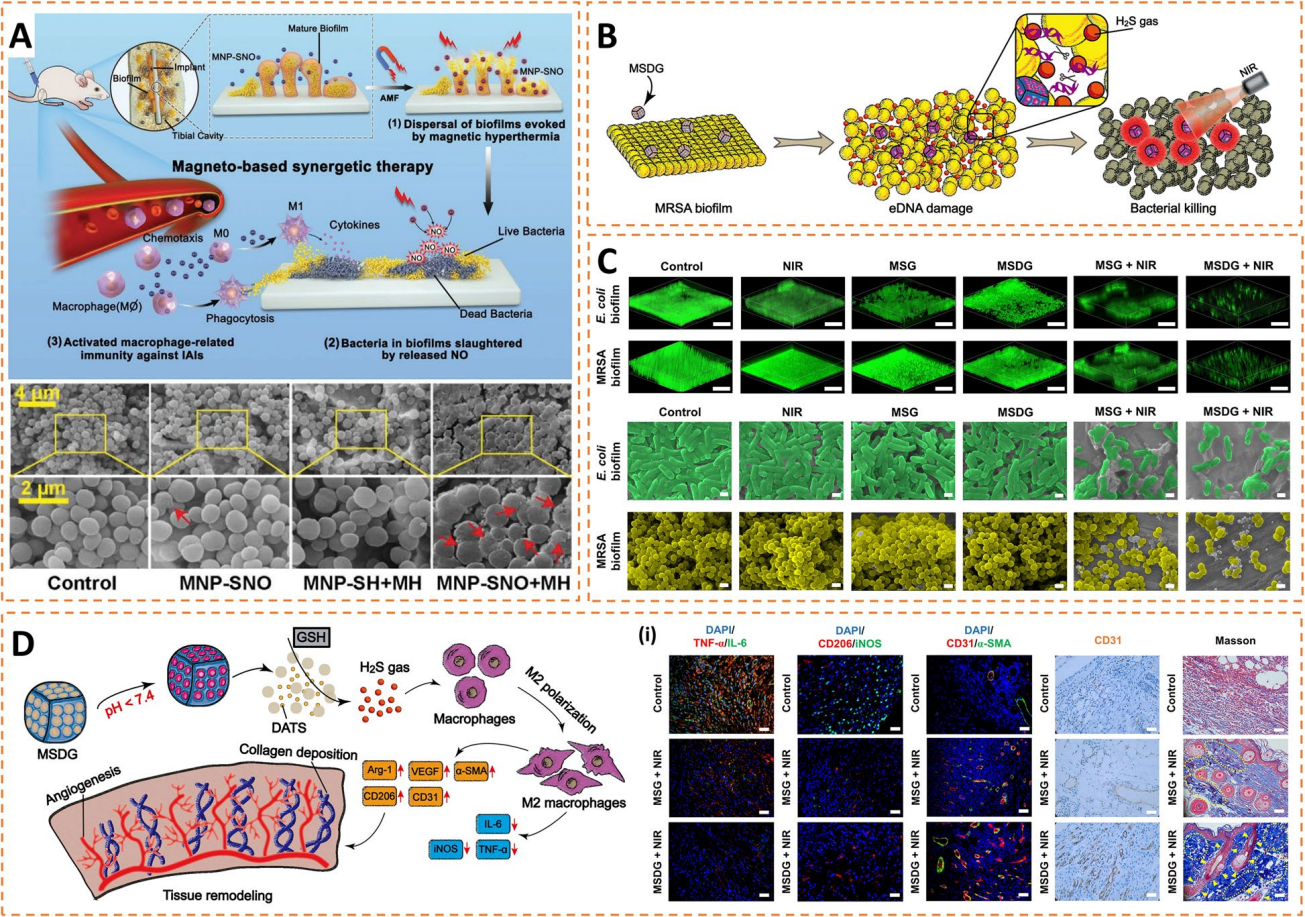
Gasotransmitters combined with magnetothermal/photothermal therapies for biofilm eradication and tissue remodeling. **A** Schematic illustration of magneto-based synergetic therapy for the eradication of bacterial biofilms. SEM images show that the synergistic therapy resulted in biofilm dispersion and morphology distortion of *S. aureus* (Control, MNP-SNO, MNP-SH + MH and MNP-SNO + MH, respectively). Reproduced with permission [104]. Copyright 2021, Wiley-VCH GmbH. **B** Antibacterial illustration of the “gas-sensitized hyperthermia” strategy. **C** Efficacy in the destruction of *E. coli* and MRSA biofilms using the “H_2_S-sensitized hyperthermia” strategy (Up: confocal microscopy images of biofilm staining; down: SEM images of biofilms). **D** Schematic illustration of H_2_S gas release reversing the proinflammatory microenvironment and promoting wound healing. (i) Immunofluorescent (TNF-α/IL-6, CD206/iNOS and CD31/α-SMA, respectively), immunohistochemical (CD31) and Masson’s staining demonstrating great tissue remodeling achieved by H_2_S therapy (Up: control; middle: MSG + NIR; down: MSG + NIR). Reproduced with permission [110]. Copyright 2022, American Association for the Advancement of Science

CO, NO, and H_2_S are important gasotransmitters with multiple biological effects [191, 192]. Recently, gasotransmitters have received much attention for their broad-spectrum antimicrobial activity. Their antibacterial effect arises from inhibition of the respiratory chain of bacteria, promotion of intracellular oxidative stress, and damage to DNA [104, 110, 193]. More importantly, these gases can easily penetrate the biofilm to kill bacteria. Gas therapies are often used in combination with other treatments (such as photothermal and magnetothermal therapies) to exhibit powerful antibacterial (such as MRSA) and anti-biofilm effects (Fig. 7 A) [104].

In addition, these gas molecules have been found to have anti-inflammatory properties, which facilitate reducing unnecessary inflammatory damage when fighting infections. For example, a “gas-sensitized hyperthermia” strategy has been proposed by Su et al. [110]. They enclosed diallyl trisulfide (a reduction-responsive donor of H_2_S) in porous Gd-doped Prussian blue nanoparticles (GPB) To prevent the leakage of drugs, MOF was used as a door seal. MOF was degraded in the acidic environment of the biofilms, releasing diallyl trisulfide to produce H_2_S in reaction with the overproduced GSH in biofilms rapidly. Aided by the heat flow generated by NIR-illuminated GPB, biofilms were eradicated through H_2_S-induced extracellular DNA damage and heat-mediated bacterial death (Fig. 7B, C). In experiments in vivo, H_2_S induced M2 polarization of macrophages, accompanied by the production of regeneration-related cytokines. This process helped reverse the infection-induced pro-inflammatory microenvironment to a regenerative environment, effectively promoting tissue regeneration (Fig. 7D). Similarly, Zhang et al. proposed an interfacial functionalization strategy by integrating carbon monoxide gas (CO) nanogenerators on titanium surfaces, followed by the covalent graft of arginine glycine-aspartic-acid (RGD) polypeptides [194]. Under near-infrared light (NIR) irradiation, the functionalized surface showed a powerful killing effect on MRSA via CO-enhanced mild photothermal therapy. Furthermore, on-demand CO delivery mediates anti-inflammatory effects by promoting heme oxygenase (HO-1) expression and inducing down-regulation of NF-κB (p50/p65) and p38 mitogen-activated protein kinase (MAPK). More importantly, the combination of released CO and immobilized RGD manipulates the reprogramming of macrophages to anti-inflammatory M2-phenotype polarization by a potential JAK1/STAT6 pathway, thereby remodeling the damaged microenvironment to a pro-regenerative environment. In a rat model of IAI, the designed implant effectively clears residual bacteria, reversing the harmful pro-inflammatory environment, achieving good osteogenesis.

Although the robust antimicrobial potential of gasotransmitters has been reported by many studies, the inaccurate delivery and dosage control of gas are extremely challenging problems. When the gas cannot be released specifically in the infected area or when the released dosage of gas is uncontrollable, it may fail to kill bacteria or even cause biotoxicity. Therefore, the development of controllable delivery has become imperative.

### Smart drug delivery strategies

Various antibacterial drugs (such as metal nanoparticles and antimicrobial peptides) have been developed to eradicate IAI. However, inappropriate degradation or burst release of drugs has been challenging. Improper degradation of the drug results in failure to achieve effective local drug concentrations to control infection. Burst release of the drug often results in toxicity and subsequent lack of residual drug dosage. The high surface area of ​​carbon-based materials and the porous structure of hydrogels have potential advantages for drug delivery. Smart release of loaded drugs can be achieved through appropriate modification of biomaterials, thereby minimizing off-target effects. In addition, both materials possess antimicrobial properties. In this chapter, we will discuss the antibacterial properties of carbon-based materials and hydrogels and their potential as antibacterial drug carriers.

#### Carbon-Based nanomaterials

Carbon-based nanomaterials (CNMs) include carbon nanotubes, graphene, mesoporous carbon, and their complexes. These materials have gradually attracted attention in the medical field due to their strong antibacterial properties, relatively low toxicity, and easy surface modification [201–203]. Their potential antimicrobial mechanisms follow various patterns, such as mechanical damage to cell membranes, oxidative stress, lipid extraction, denutrition, inhibition of bacterial metabolism, and photothermal or photocatalytic effects [201, 204]. CNMs can also inhibit biofilm formation by inhibiting bacterial adhesion, interfering with the sensing system, and other means [205]. Therefore, CNMs have great potential for antimicrobial applications.

**Fig. 8:**
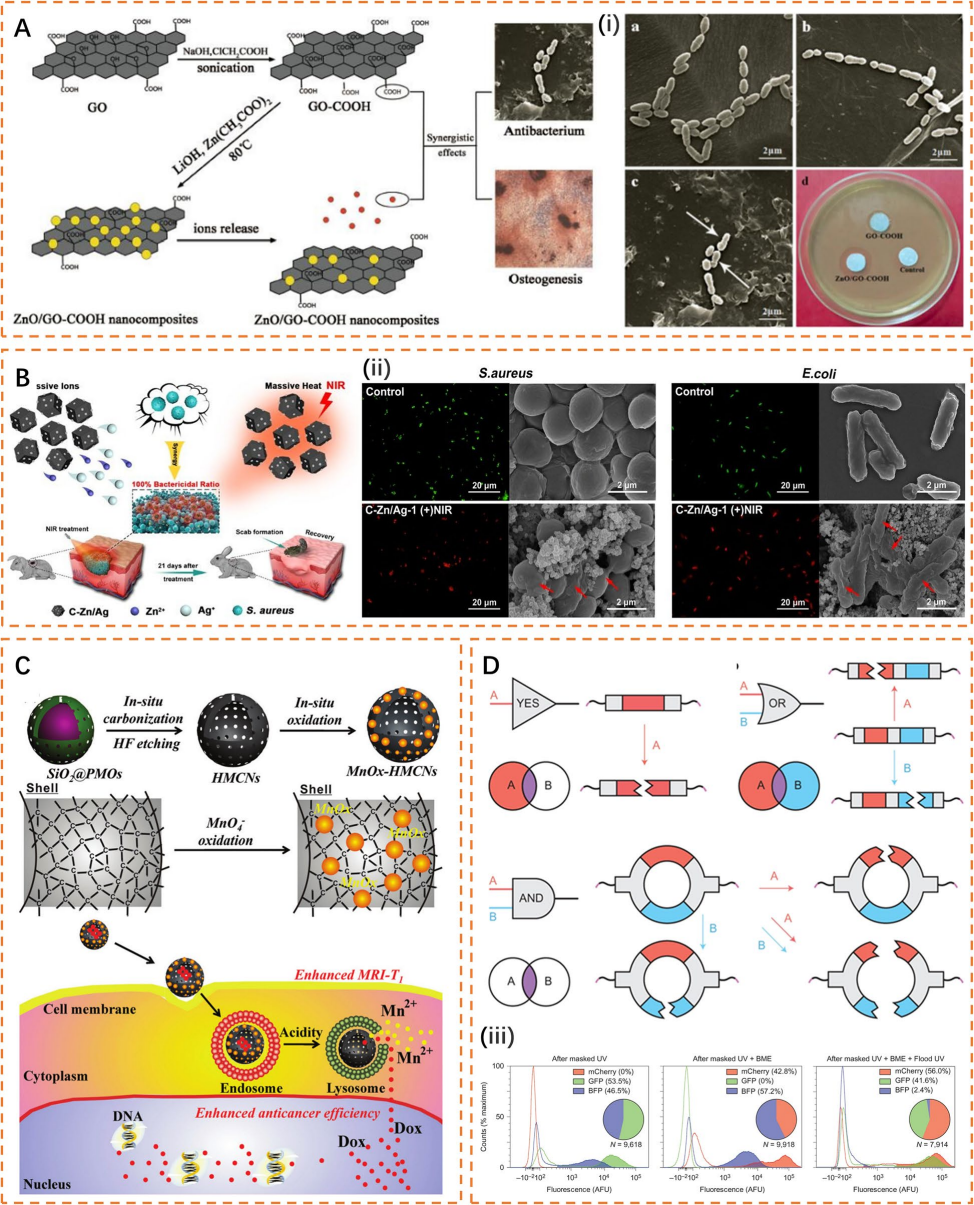
**A** Schematic illustration of the synthesis of ZnO/GO-COOH nanocomposites and action mechanisms for the synergy of antibacterial and osteogenic effects. (i) SEM images and disk diffusion assay showing the antimicrobial activity of ZnO/GO-COOH nanocomposites against *E. coli*. Reproduced with permission [206]. Copyright 2016, Elsevier B.V. **B** Schematic image illustrating the synergistic bacterial eradication mechanism based on NIR-induced hyperthermia and metal ion release from C-Zn/Ag-derived nanocomposites. (ii) Live/dead staining and SEM images indicating the bactericidal efficacy of nanocomposites combined with NIR. Reproduced with permission [105]. Copyright 2020, American Chemical Society. **C** Scheme illustrations of the synthesis of mesoporous carbon nanoparticles doped with manganese oxides and dual pH/ultrasound responsiveness for anticancer drug release. Reproduced with permission [106]. Copyright 2015, Royal Society of Chemistry. **D** Scheme illustrations of rationally designed crosslinker architecture to achieve stimuli-responsive biomaterial degradation. (iii) Flow cytometry quantification of cells released from hydrogels in response to environmental cues. Reproduced with permission [225]. Copyright 2018, Springer Nature

The high surface area and easy surface modification of CNMs make them excellent carriers. Chen et al. synthesized a ZnO/GO-COOH nanocomposite (Fig. 8 A) [206]. ZnO nanoparticles were easily decorated on graphene oxide by carboxylation of graphene oxide (GO) and subsequent nucleation of ZnO on GO-COOH sheets. This nanocomposite retained the antimicrobial activity and osteogenesis of the original material. Yang et al. took full advantage of the carrier capability and photothermal conversion properties of CNMs. They introduced Zn^2+^ and AgNPs into a graphite-like carbon skeleton to fabricate C-Zn/Ag-derived nanocomposites (Fig. 8B) [105]. Under near-infrared radiation, the nanocomposites can generate a large amount of heat to destroy the bacterial film. Furthermore, massive amounts of Ag^+^ and Zn^2+^ are released to cause chemical damage to bacteria. In antimicrobial experiments in vitro, this synergistic antibacterial effect exhibited nearly 100% bactericidal activity against bacteria at an extremely low dose (0.16 mg/ml). Zhang et al. integrated manganese oxide nanoparticles into hollow mesoporous carbon nanoparticles by an in situ framework redox technique. The material achieved pH and ultrasound triggered the release of drugs based on the “fragmentation” properties of the manganese oxide and the interaction of the carbon backbone with the drugs (Fig. 8 C) [106]. More of these multi-stimulus-responsive nanosystems are expected to be used in anti-infection applications.

Titanium alloy is a common biomaterial in medical devices. CNMs can be used to coat titanium substrates by easy fabrication processes, such as electroplating, micro-arc oxidation and the layer-by-layer (LbL) approach [207–209]. For instance, Nie et al. fabricated a self-sterilizing and biocompatible surface film coating by using polymer-shielded silver nanoparticle-loaded oxidized carbon nanotube (AgNPs@oCNT) nano-dispersions [207]. Specifically, the coating was produced by alternately depositing bioinspired positively charged and negatively charged AgNPs@oCNTs on substrates by LbL. The AgNPs@oCNTs coating exhibited long-lasting efficient killing towards bacteria in multiple experiments. Simultaneously, the coating showed remarkable blood compatibility and limited toxicity to mammalian cells due to the shielding effect of the polymer layers. Based on the photothermal and photocatalytic properties of carbon materials, Li et al. developed a Nitrogen-doped carbon dots (NCDs)/ Hydroxyapatite (Hap) modified Graphene oxide (GO) heterojunction film on Ti substrates for a mild phototherapy nanoplatform, which showed increased electron-hole pair separation and inhibited recombination efficiency via hole depletion [210]. The metabolism of bacteria on this film was significantly inhibited under light irradiation because of the enhanced photothermal and photocatalytic effects. Moreover, electron transfer from the transmembrane protein complex of *S. aureus* to the GO/NCD/Hap/Ti film further inhibited the ATP synthesis process. More interestingly, the consequent photocurrent induced Ca^2+^ flow for cell adhesion and migration and tissue reconstruction. Meanwhile, the promoted inflammation associated with the M1 polarization of TNF-α and IL-6 upregulation and induced CD4+/CD8 + lymphocytes was ameliorated in the injured tissues by activating the PI3K/P-AKT pathway. This mild phototherapy combining sterilization and immunomodulation will be promising for a noninvasive and safe therapeutic strategy.

Despite the promising progress in the exploration of CNMs as antimicrobial agents, it is still too early to apply CNMs in practical applications to replace existing antimicrobial materials (such as antibiotics and antimicrobial peptides). First, the biological toxicity of CNMs remains a concern. Previously, pulmonary fibrosis induced by carbon nanotube exposure was observed in rodent animals due to oxidative stress and inflammatory response [211, 212]. Some studies have observed that pristine carbon nanomaterials lead to apoptosis or necrosis of immunocytes by inducing intracellular production of ROS or mitochondrial dysfunction [213–215]. These carbon nanomaterials that cause cytotoxicity are often unmodified by functionalization (i.e., pristine carbon nanomaterials). Several studies demonstrated the significant reduction in biotoxicity after the functionalization of CNMs [105, 216, 217]. In addition, toxicity data reported in most studies were observed under high-dose or acute-exposure conditions, which were not helpful for the understanding of their immunomodulatory mechanisms. Kinaret et al. explored the effect of different types of CNMs on phenotypic changes in macrophages at non-lethal doses (10 µg·mL^− 1^) [218]. After 48 h of exposure, they observed that macrophages could self-regulate and change their signal cascade reactions in response to different CNM exposures and then differentiate toward different phenotypes. The results suggested that even harmful nanomaterials could exert immunomodulatory effects by careful dose adjustment. Interestingly, Svadlakova et al. studied the impact of carbon nanotubes and GPs (two common pristine CNMs) on human primary monocytes [219]. They found that CNMs caused neither direct cytotoxicity nor proinflammatory cytokine release. In contrast, carbon nanomaterials promoted monocyte-mediated phagocytosis and the release of cytokines (TNF-α, IL-6, and IL-10) in the presence of bacteria. This confirmed the immunomodulatory ability of the carbon nanomaterials.

In addition, the lack of methods for exact preparation is also a limitation. Although a variety of methods have been developed to fabricate CNMs, CNMs produced by different methods or even the same method from different precursors are usually observed to have different antibacterial performances. For example, graphene quantum dots (GQDs) prepared by rupturing a C60 cage can effectively kill *S. aureus*, whereas those prepared from graphene oxide sheets show no antibacterial properties [220]. Furthermore, impurities in the manufacturing of CNMs (such as metal catalysts or amorphous carbon in carbon nanotubes) can also interfere with the accurate estimation of their antimicrobial properties, which may be related to the nonstandard and complex preparation process [221, 222]. In view of these challenges, developing economical and green approaches for the large-scale synthesis of CNMs with high reproducibility is extremely desirable. Despite these obstacles, CNMs are still considered promising for antimicrobial applications in the future.

#### Hydrogel

Hydrogels are cross-linked macromolecular networks that can retain water extensively [223, 224]. It has inherent advantages as a carrier of cells or drugs. The biophysical properties of the hydrogel are quite similar to the extracellular matrix, which is conducive to the penetration and growth of endogenous cells. The porous properties of hydrogels allow nutrients and metabolic waste to pass through freely. As drug carriers, hydrogels can prevent the premature degradation or burst release of drugs. By changing the pore size and tightness of the hydrogels, the release speed of drugs can be regulated. Rational hydrogels are constructed for the smart responsive release of drugs [224–227]. Badeau et al. developed a modular chemical framework to endow hydrogels with degradation responsive capability to diverse environmental cues such as enzymes, reducing agents, and lights (Fig. 8D) [225]. This precise responsiveness to the environmental cues is obtained by altering the specific stimuli-labile moieties of crosslinkers in hydrogels.

**Fig. 9:**
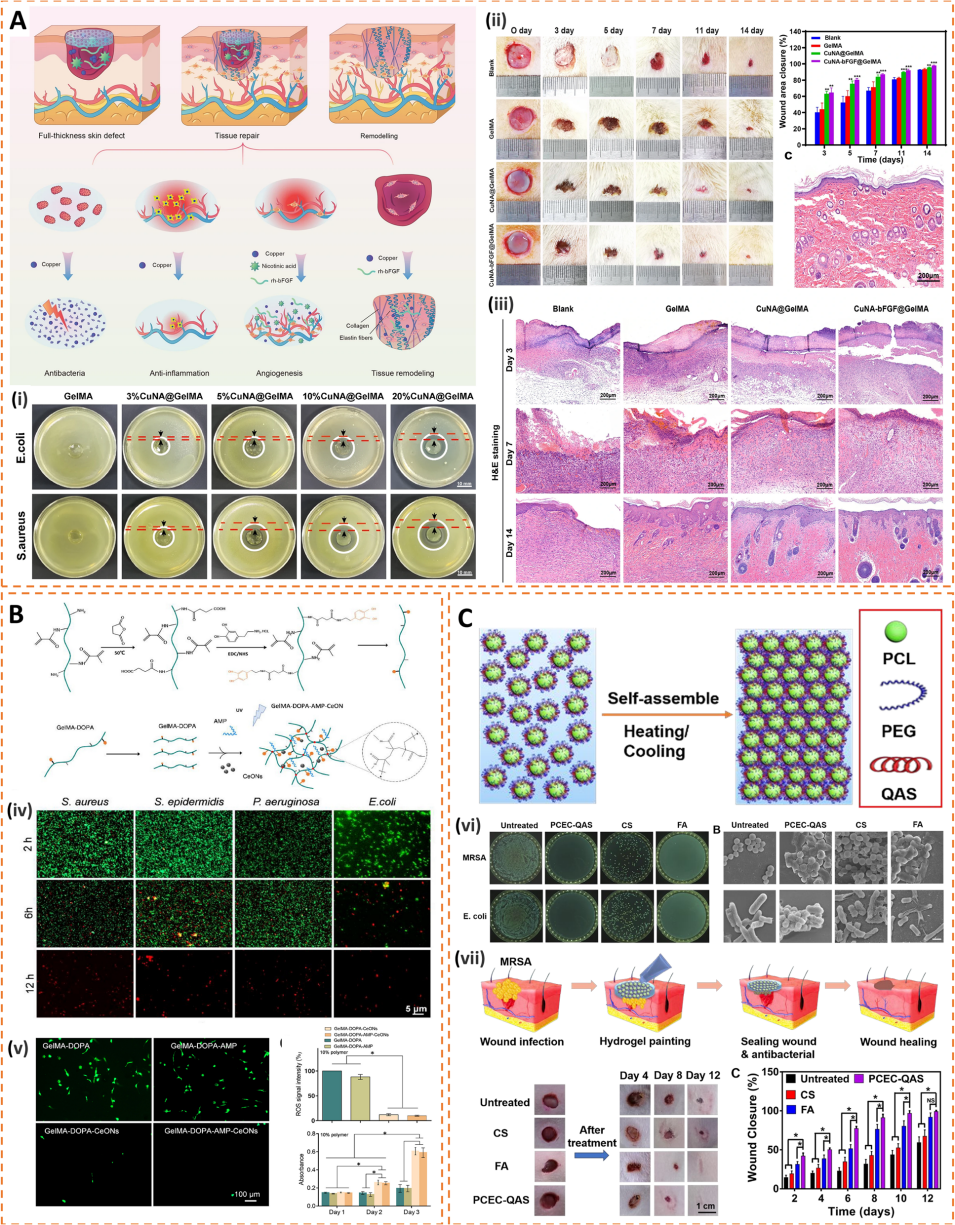
Drug delivery vehicles and self-assembled hydrogels for antibacterial and wound healing applications. **A**. Schematic image illustrating the fabrication of composite hydrogels coloaded with copper and bBGF for antibacterial activity and tissue remodeling. (i) Inhibition zone tests to compare antibacterial effects of the hydrogel loaded with different concentrations of copper‑nicotinic acid. (ii) (iii)Optical and H&E staining images demonstrated that the hydrogel accelerated the process of wound healing. Reproduced with permission [108]. Copyright 2021, Springer Nature. **B**. Schematic illustration of process involved in the synthesis of GelMA-DOPA hydrogel loaded with AMP and CeONs. (iv) Live/dead staining images showing the antibacterial abilities of the AMP-loaded hydrogel to different bacteria species. (*S.aureus, S.epidermidis, P.aeruginosa* and *E.coli, respectively*) (v) Fluorescence images and quantitative analysis of ROS formation showing the ROS-scavenging ability of CeONs. Reproduced with permission [228]. Copyright 2021, Elsevier. Ltd. **C**. Schematic illustration of the structure of PCEC-QAS nanoparticles that self-assembled into hydrogels. (vi) Optical and SEM images showing the antibacterial efficacy of the PCEC-QAS hydrogel. (vi) Tissue repair processes after painting with different hydrogels (Untreated, CS, FA and PCEC-QAS hydrogel). Reproduced with permission [230]. Copyright 2020, American Chemical Society

The hydrogels can be endowed with good antibacterial and immunomodulatory activities by incorporating bioactive components. Wang et al. prepared a composite hydrogel loaded with copper and growth factors (basic fibroblast growth factor, bFGF) (Fig. 9 A) [108]. Due to the synergistic effects of copper and bFGF, the composite hydrogel not only exhibited excellent antibacterial activity but also promoted cell migration and angiogenesis activity. In rat models of full-thickness cutaneous deficiency, the prepared hydrogel significantly inhibited the inflammatory response and promoted neovascularization and deposition of elastic fibers and collagen, which accelerated wound healing. Inspired by the adhesion of mussels, Cheng et al. developed a hydrogel conjugated with dopamine (DOPA) to enhance adhesion to wound surfaces (Fig. 9B) [228]. An antimicrobial peptide (AMP) and cerium oxide nanoparticles (a ROS scavenger) were further encapsulated into the hydrogel to impart its antibacterial and ROS abilities. The prepared hydrogel dressings have the advantages of adhesiveness, antibacterial activity, ROS scavenging, and skin remodeling abilities.

Some antimicrobial materials (such as polysaccharides and peptides) can be formed into hydrogels, which retain the original antimicrobial properties and greatly simplifies the fabrication process of biomaterials [229, 230]. Liu et al. developed an inherently antibacterial and bioresorbable hydrogel by conjugating a modified quaternary ammonium salt (QAS) with poly(ε-caprolactone)-poly(ethylene glycol)-poly(ε-caprolactone) (PCEC) for PCEC-QAS. Amphiphilic PCEC-QAS can self-assemble into nanoparticles (NPs) in water and subsequently self-aggregate to form hydrogels after heating-cooling treatment (Fig. 9 C) [230]. In vivo experiments showed that the hydrogels not only exhibited intrinsic antibacterial effects but also accelerated skin regeneration at the infected wound in the absence of antibiotics or cytokines. The self-assembly of such amphiphilic adducts provides a facile method for the preparation of antimicrobial agents with intrinsic antimicrobial ability. Wahid et al. used the complexation reaction of biopolymers with transition metal ions (such as Ag+, Cu^2+,^ and Zn^2+^) to rapidly fabricate antimicrobial hydrogels [231]. They mixed carboxymethyl chitosan solution (CMCH) with a metal salt solution of suitable pH value to prepare the hydrogel. FTIR measurements indicated that rapid hydrogelation was promoted by the simple complexation of metal ions with carboxyl, amino, and hydroxyl groups of CMCH. Hydrogels show not only stronger antibacterial properties but also remarkable plasticity.

Hydrogel coatings can be constructed on the surface of implants to exert antimicrobial and immunomodulatory effects in situ. On the one hand, the similarity of the hydrogel to physiological tissues can guide cellular penetration at the initial stage of implantation and promote biological adhesion of the implant to the host. On the other hand, the hydrogel coating can maintain the durable stability of the bioactive agents loaded on the underlying layer and allow their controlled release. However, the weak bonding between hydrogels and substrates usually severely hinders their integration and function in implants. To solve this problem, Yuk et al. developed a strategy to covalently immobilize hydrogel coatings on nonporous solid substrates by simple silane modification of target surfaces [232]. Compared with physical interactions, such chemical bonding achieved a higher intrinsic work of adhesion (over 1000 J m^− 2^), superior to the toughness of cartilage-bone and tendon-bone interfaces (interfacial toughness∼800 J m^− 2^). Inspired by the ability of mussels to adhere to nearly any surface under water, Chen et al. grafted catechol motif onto chains of methacrylated gelatin to develop an adhesive multifunctional hydrogel [233]. Moreover, antimicrobial peptides (AMPs) and osteoconductive silicate nanoparticles (SNs) were physically loaded into this hydrogel to achieve controlled delivery postoperatively. The tough coordination bonds between the catechol motifs and Ti substrates could further enhance the binding strength. Meanwhile, the introduction of AMP and SNs endowed hydrogels with extraordinary antimicrobial and osteoconductive properties.

Similarly, He et al. constructed a dual drug-loading system on titanium implants by adhering catechol-motif-modified methacrylated gelatin (GelMA) hydrogel to TiO_2_ nanotubes [234]. The formation of porous TiO_2_ nanotubes on the Ti surface by anodic oxidation not only enhanced the adhesion between the hydrogel coating and the Ti substrate, but also served as a storage reservoir for sequential drug release. Specifically, they loaded IL-4 into nanotubes and embedded CaO_2_ nanoparticles into the hydrogel seal to achieve both antibacterial and anti-inflammatory properties. First, CaO_2_ nanoparticles embedded in hydrogels rapidly eliminated bacteria by releasing H_2_O_2_. Subsequently, the gradual release of IL-4 loaded in the nanotubes alleviated the pro-inflammatory response induced by bacteria and CaO_2_ and promoted the M2 polarization of macrophages. In the murine infection model, *S. aureus* contamination was effectively eliminated after 2 days, and the accompanying excessive pro-inflammatory response was also suppressed within 1 week. Finally, the damaged tissue recovered significantly. This dual drug delivery system will provide a promising strategy for implant modification with multiple physiological properties.

### Limitations of immune-enhanced antimicrobial strategies

There are still many limitations that prevent these antimicrobial strategies from bench to bedside. The biotoxicity of biomaterials is the primary concern. The biotoxicity of metal nanoparticles and carbon-based nanomaterials has been widely discussed [215, 235]. The specific mechanisms involve the disruption of cell membranes, induction of oxidative stress, and DNA damage. Although some clinical trials using gas therapy are ongoing, widespread applications of gasotransmitters present certain challenges due to difficulties in dosage control and deficiency of tissue specificity [236]. HDPs are derived from living organisms and are considered to have low toxicity. However, it is possible to produce unwanted off-target effects other than anti-infection effects due to their extremely wide biological activity. Sensitivity to proteases and high cost are also barriers to their application. Various strategies have been mentioned above to enhance the stability of antimicrobial peptides, including replacement of L-amino acids with D-amino acids, chemical modification of peptides to enhance protease resistance, development of antimicrobial peptide analogs, and use of transported carriers (such as liposomes and hydrogels). However, there is a lack of assessment of the activity, longevity and toxicity of these modified antimicrobial peptides.

Some proven antimicrobial agents, such as metal nanoparticles and HDPs, have been reported to have the risk of counteracting normal host defenses and promoting the development of infection when applied in inappropriate doses. In fact, for most immunomodulatory drugs, there is only a narrow therapeutic window between antimicrobial and toxic doses. Different investigators have reported conflicting results on the effective dose of drugs. In this regard, it is necessary to define uniform experimental criteria and carefully design in vivo studies to obtain reliable experimental results in the future.

Another emerging challenge is the development of drug resistance. Metal nanoparticles and HDPs are considered to have a low propensity for drug resistance previously. Unfortunately, some studies have observed bacterial resistance to these biomaterials. Resistance to metal nanoparticles may involve the promotion of metal nanoparticle aggregation and the upregulation of metal ion efflux genes [237, 238]. The main pathways mediating bacterial resistance to antimicrobial peptides include increased efflux, proteolytic degradation, and membrane modifications. The mechanism of antimicrobial activity of these biomaterials and the mechanism of bacterial resistance still deserve to be explored in depth. This is important to guide the future large-scale application of these novel biomaterials, as we have made such mistakes in the abuse of antibiotics before.

## Conclusions and future perspectives

Implant-related infection is a serious clinical problem. Biomaterials provide anchors for bacteria and inhibit the normal function of immune cells. The currently used prosthetic materials are generally not antimicrobial. Unreasonable antibiotic therapy in the clinic has spawned the rapid development of bacterial resistance. A deeper understanding of the genetic basis of bacterial resistance is significant for the development of novel antimicrobial strategies. Applications of new technologies may help in relentless efforts to fight bacteria. For example, next-generation sequencing technologies have facilitated the rapid identification and characterization of antibiotic resistance genes in the genome [239]. The development of machine learning algorithms has made it possible to predict bacterial resistance phenotypes and molecules with antimicrobial activity [240]. Future studies on antimicrobial resistance require multidisciplinary efforts.

The innate immune system has a significant role in defense against infection. Current exogenous antimicrobial therapies, such as antibiotics, mainly emphasize the direct killing of bacteria while ignoring the regulation of immune cells. The ideal anti-infection strategy should not only have a significant bactericidal effect but also activate immune cells to kill the remaining bacteria. The mechanisms of immune cells, especially neutrophils, fighting infections are still not clear. As the most abundant leukocytes in the circulation, neutrophils have a rapid response to infection. However, it may be underappreciated due to the lack of studies on its application in the field of biomaterials. The in-depth exploration of the function of neutrophils is hampered because they have a very short lifespan (5–90 h) and no cell line greatly reflects the functions of neutrophils [241, 242]. Studies of neutrophils in vivo also raise concerns. Mouse neutrophils are the model of choice for in vivo studies. However, the proportion of neutrophils in mice is significantly different from that in humans (25% versus 70% in mice and humans, respectively) [243]. This makes the conclusions from in vivo studies should be interpreted with caution. Furthermore, there are many challenges in understanding the functions of other immune cells, such as MDSCs. More detailed performance of these immune cells in anti-infective strategies should be investigated in the future.

Given the rapid development of bacterial resistance and the growing understanding of the immune system defending against infection, immune-enhanced antimicrobial strategies are considered promising for the treatment of IAI [13, 244]. A variety of nanomaterials have been found to significantly affect the behavior of immune cells and bacteria. Indeed, some nanomaterials have been used for clinical practice. The most representative of them are silver nanoparticles, which are widely used as antimicrobials in medical, industrial and domestic applications, such as supplies, wound dressings and medical devices [245]. In the field of orthopedics, several clinical series of silver-coated megaprosthesis have been reported, resulting in reduced infection rates [246–248]. Wafa et al. reported a lower incidence of infection with silver-coated megaprosthesis (11.8%) compared with traditional megaprosthesis (22.4%) [246]. Eto et al. first reported clinical experience with silver containing hydroxyapatite (Ag-HA) coating implants [249]. In this prospective interventional study, they performed THA with such implants in 20 patients to examine the merit of silver. During the one-year follow-up period, the patient’s blood silver was always within the safe range. Activities of daily living were significantly improved in all cases. No implant failure was observed on radiographs. None of the patients developed infection or adverse reactions to silver after operation. Silver-coated implants seem promising in preventing IAI according to the present studies. In the future, randomized controlled trials with larger numbers of patients and longer follow-up time are needed to verify this conclusion. In addition, several natural and synthetic HDPs have entered clinical trials seeking regulatory approval for use as anti-infective agents [172, 250]. Although most non-ribosomal peptides have not yet received regulatory approval for clinical application, they have attracted increasing interest in their potential use in indwelling medical devices and may be championed for the prevention of resistance emergence and to eliminate intractable disease that traditional antimicrobial agents have failed [168, 251].

Potential cytotoxicity, high cost and unreliable antimicrobial efficacy in vivo are the main factors limiting these novel strategies from bench to bedside. Developing low-cost and green approaches for manufacturing these immunomodulatory agents is highly desirable. Several smart delivery systems have been explored for the controlled release of immunomodulatory agents to optimize the antimicrobial properties of materials and reduce toxicity. Even so, the response of cells to these novel biomaterials should still be carefully scrutinized for safety. The property parameters of these materials also need to be optimized for the desired immune effects. Recently, high-throughput technologies have become increasingly popular for the screening and validation of bioactive materials [252, 253]. The standardized technical process minimizes experimental bias and is more cost-effective. Artificial intelligence can also be used for the analysis of experimental data to predict the response of immune cells to biomaterials and for improving the design of materials [254]. The boom of these technologies has opened up possibilities for the rational design of biomaterials.

## Data Availability

Not applicable because this is a review article and no data were newly generated.
